# Tracking the HS-HS, HS-LS, and LS-LS States in the Spin Transition of a Dinuclear Fe(II) Complex by Broadband FTIR Spectroscopy

**DOI:** 10.1021/acs.jpclett.5c03119

**Published:** 2025-12-05

**Authors:** Marcel Walter, Eike F. Kuhlemann, Tarek Al Said, Clara W.A. Trommer, Felix Tuczek, Karsten Holldack, Wolfgang Kuch, Sangeeta Thakur

**Affiliations:** † Institut für Experimentalphysik, 9166 Freie Universität Berlin, Arnimallee 14, 14195 Berlin, Germany; ‡ Institut für Anorganische Chemie, 9179 Christian-Albrechts Universität zu Kiel, 24098 Kiel, Germany; ¶ 28340 Helmholtz-Zentrum Berlin für Materialien und Energie GmbH, Hahn-Meitner-Platz 1, 14109 Berlin, Germany

## Abstract

Highly resolved vibrational spectra of the mononuclear Fe­(II) spin-crossover complex [Fe­(bpz)_2_(bipy)]; (bpz = dihydrobis­(pyrazolyl)­borate) and its dinuclear counterpart [{Fe­(bpz)_2_}_2_μ–(ac­(bipy)_2_)] (ac­(bipy)_2_ = bridging ligand) are obtained by temperature-dependent far-infrared (FIR) spectroscopic measurements and assigned with the help of density functional theory (DFT) calculations. The experimental data confirm the high-spin (HS) state of the complexes at high temperature (≈ 200 K) and the low-spin (LS) state at 5 K. In the dimer, enhancement of otherwise absent vibrational modes at 335 cm^–1^ and 504 cm^–1^ around *T*_1/2_ reflects the presence of a mixed-spin HS-LS state during the course of the spin transition between HS-HS and LS-LS. The metastable HS state of the dinuclear complex resulting from light irradiation (532 nm) at 10 K results from a direct spin-state transition from LS-LS to HS-HS.

## Graphical abstract

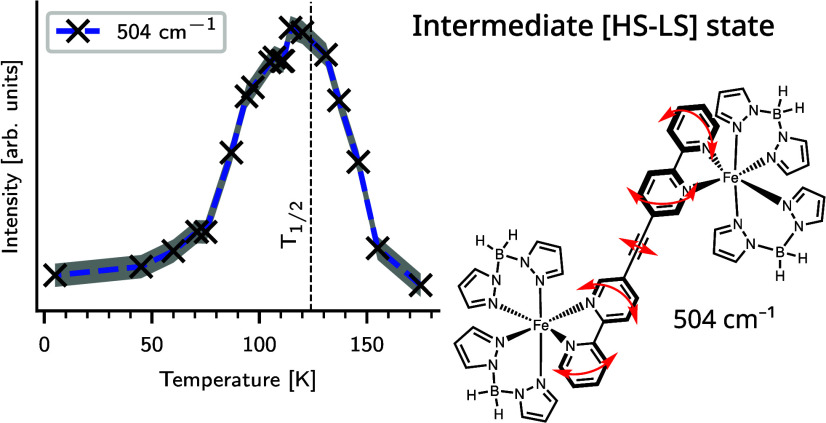

The spin-crossover (SCO) phenomenon is observed in transition metal ions of configuration d^4^ to d^7^ and can be controlled by external stimuli such as temperature, light, electric field, or pressure,^1–11^ giving rise to interesting applications such as molecular electronics, data storage, and display devices.^12–19^ The ligand-field strength around the transition-metal ions affects the energetic splitting of the d shell into t_2*g*_ and e_*g*_ levels, and a different population of these levels leads to HS and LS states of the complex (Figure 1c). Most of the studies on SCO complexes have been conducted under temperature variation and/or irradiation with light.^3–6^ The thermal spin transition is an entropy-driven process; i.e., the higher spin multiplicity and the higher density of vibrational states favor population of the HS state at elevated temperatures. Alternatively, a transition from the LS ground state to an excited HS state can be effected by irradiation with light (light-induced excited spin-state trapping; LIESST).^20^

**Figure 1:**
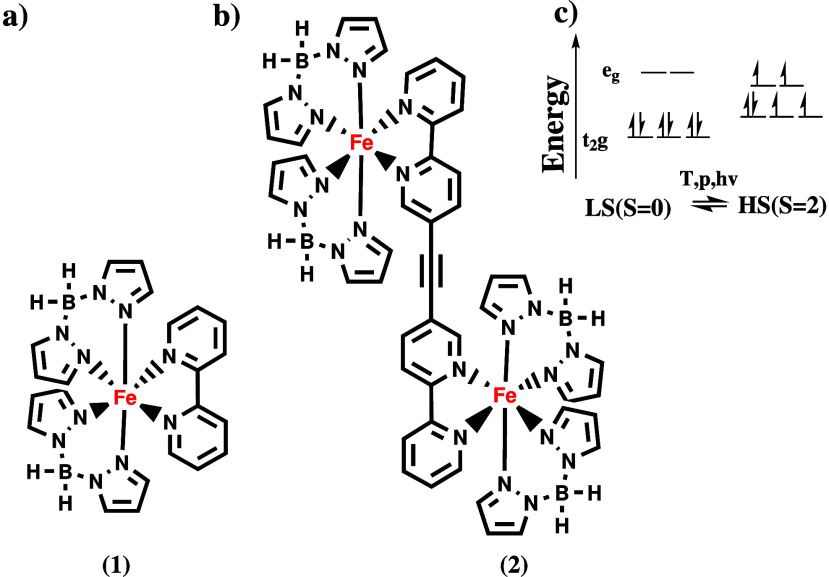
Structure of (a) the mononuclear complex [Fe­(bpz)_2_(bipy)] (**1**) with one Fe (II) center, (b) the dinuclear complex [{Fe­(bpz)_2_}_2_μ–(ac­(bipy)_2_)] (ac­(bipy)_2_ = bridging ligand) (**2**) with two monomers bridged by an acetylene, and (c) schematic energy diagrams of the octahedral ligand field for the Fe­(II) ion in two possible spin states and possible perturbations to obtain spin-crossover.

The investigation of spin-crossover complexes involves a broad range of methods that can be applied to gain information about different physicochemical properties of the sample, including measurements of magnetic susceptibility, X-ray crystallography, and UV/vis- and Mößbauer as well as Raman spectroscopies.^21–26^ A typical structural motif of these compounds is the Fe­(II)-N_6_ core, which is contained in many SCO complexes. Vibrational information about this moiety is most easily inferred from the spectral region below 500 cm^–1^. This part of the spectra is most diagnostic with regard to the spin state as it directly reflects the shift of metal–ligand vibrations that occurs due to alteration of the Fe–N-bond lengths upon SCO. The spin-state dependence of the intramolecular vibrational modes thus can be understood in detail by combining state-of-the-art vibrational spectroscopies (IR) with DFT calculations.^27^

There have been many studies on mononuclear Fe­(II) SCO complexes in the bulk, mostly directed toward elucidating the origin and possible tuning of cooperative effects.^9–11,28^ Moreover, Fe­(II) SCO complexes have been deposited as thin films on different substrates and investigated with a range of surface-spectroscopic and -analytical methods.^3–6,29–31^ A few of these studies have also been conducted on dinuclear Fe­(II) SCO complexes, where two metal centers are linked to each other by bridging ligands (Figure 1b).^23,32–36^ The temperature- or light-induced spin-state transitions of these systems are intrinsically more complex than those of their mononuclear counterparts due to the possibility of one of the Fe centers switching from HS to LS or vice versa, with the other Fe center remaining in its initial state. This behavior may result in a stepwise spin-state transition upon variation of temperature or irradiation with light.^33–36^ If the spin-state transition passes through a mixed [HS-LS] state as a function of temperature, new vibrational modes characteristic of this state should appear in the far-infrared (FIR) spectrum. These extra bands are distinct from the bands observed for the HS-HS and LS-LS states at high and low temperature, respectively. An example of this scenario is provided by the dinuclear complex [(Fe­(bt)­(NCS)_2_])_2_ bpym], where the transition through a mixed spin-state [HS-LS] is reflected by a Raman-active mode at 1480 cm^–1^ that is fairly intense at intermediate temperature (145 K), whereas its intensity drops at 293 and 5 K.^34^ On the other hand, Nakano et al.^33^ stated that strong intermolecular interactions in the complex favor the like-spin-state pairs [HS-HS or LS-LS] over the mixed ones [HS-LS]. This was inferred from temperature-dependent FIR spectra, where an intensity change of the HS and LS modes rather than the appearance of new modes was observed.

Recently we studied the mononuclear SCO complex [[Fe­(bpz)_2_(bipy)] (**1**) and its dinuclear counterpart [{Fe­(bpz)_2_}_2_μ–(ac­(bipy)_2_)] (**2**; bpz = dihydrobis (pyrazolyl) borate, ac­(bipy)_2_ = bridging ligand, cf. Figure 1a,b) in the bulk^23^ and deposited on surfaces by pulsed layer injection.^32^ Based on magnetic susceptibility measurements, Mössbauer spectroscopy, X-ray absorption (XAS), and Raman spectroscopy, spin switching upon temperature variation and light illumination was demonstrated.^5,22,23,32^ Moreover, **1** and **2** were investigated by FIR spectroscopy,^22,23^ but the question of whether the dinuclear complex **2** undergoes spin-state switching via a mixed state, which would correspond to a HS-HS ↔ HS-LS ↔ LS-LS spin transition pathway, could not be answered due to insufficient signal-to-noise ratio and insufficient resolution of the spectra below 600 cm^–1^. Moreover, as no step was visible in the magnetic susceptibility curve^23^ in the vicinity of *T*_1/2_ [at this transition temperature, both HS and LS states are equally populated], no evidence for the occurrence of HS-LS dimers in the course of the thermal spin transition of **2** was obtained (Figure 2).

**Figure 2:**
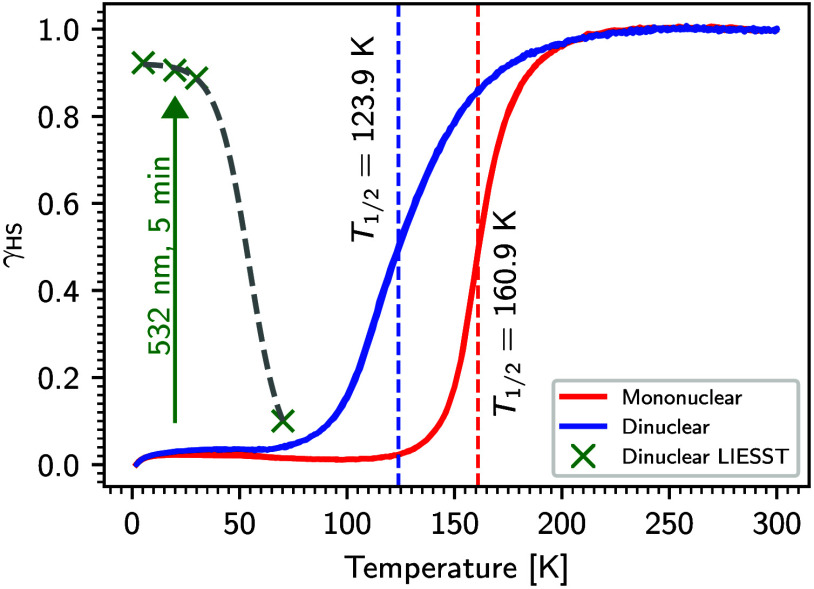
Temperature-dependent high-spin fraction (γ_HS_) of the mononuclear (**1**) and the dinuclear (**2**) complex.^23^ LIESST measurements on the dinuclear complex (**2**) using green light (532 nm) at 5 K.^23^

In order to obtain further insight into this problem, we decided to reinvestigate the vibrational properties of **1** and **2** using an optimized Fourier-transform infrared spectroscopy (FTIR) setup for both the FIR and MIR ranges.^37^ Thereby, we were able to measure vibrational spectra with high sensitivity and accuracy in the relevant range below 600 cm^–1^ as a function of the temperature and light, complemented by density functional theory (DFT) calculations. The new data now in fact provide evidence for a mixed HS-LS state that is populated in the course of the thermal spin transition of dinuclear complex **2**. Moreover, we studied the light-induced excited spin-state trapping (LIESST) of **1** and **2** by exposing the samples to green light (532 nm) at 5 K. The response of the vibrational modes to the light shows that **2** follows a direct spin-transition pathway from LS-LS to HS-HS. The implications of these findings for spin transitions in multinuclear SCO systems are discussed.

The mono- and dinuclear complex were synthesized and characterized in earlier work.^22,23,38,39^ More detailed sample preparation and measurement conditions can be found in the Supporting Information (SI). In order to assign distinctive vibrations to the observed spectral bands, the structure and vibrational modes of **1** and **2** were calculated by DFT, using Orca 6.1 ^40–42^ [details are given in the SI].

Figure 3a,b shows the FIR absorbance spectra for the mono- and dinuclear complexes **1** and **2**, respectively. Both complexes undergo a transition from HS to LS, which is evident from a change in band positions and intensities as the temperature decreases, while some signals disappear completely.^22,23^ Notably, the position of the peaks for **1** and **2** match with earlier reports by Ossinger et al. and Trommer et al., respectively.^22,23^ However, the signals are much more visible due to the higher sensitivity in the present measurements than in these publications. In order to obtain insight into the origin of the vibrational bands of monomer **1** and dimer **2**, DFT was employed. Whereas for **1** and the LS-LS configuration of **2** this has been performed before,^22,23^ we now were able to conduct the analogous calculations for the HS-HS and LS-HS configurations of **2** as well. For practical reasons the relevant vibrations in the 200 to 600 cm^–1^ region are labeled A to I, whereby roman letters refer to the HS and italic letters to the LS state (cf. Figure 3). The eigenvectors of these modes are graphically represented in Figures S1 and S2 for **1** and **2**, respectively. Thereby modes A–C are Fe–N stretching vibrations; modes D–F correspond to vibrations of the dihydrobis­(pyrazolyl)­borate (bpz) units, and modes G–H correspond to vibrational motions of the bipy moieties. For the sake of comparability, identical denominations are used for analogous modes of the monomer and the dimer.

**Figure 3:**
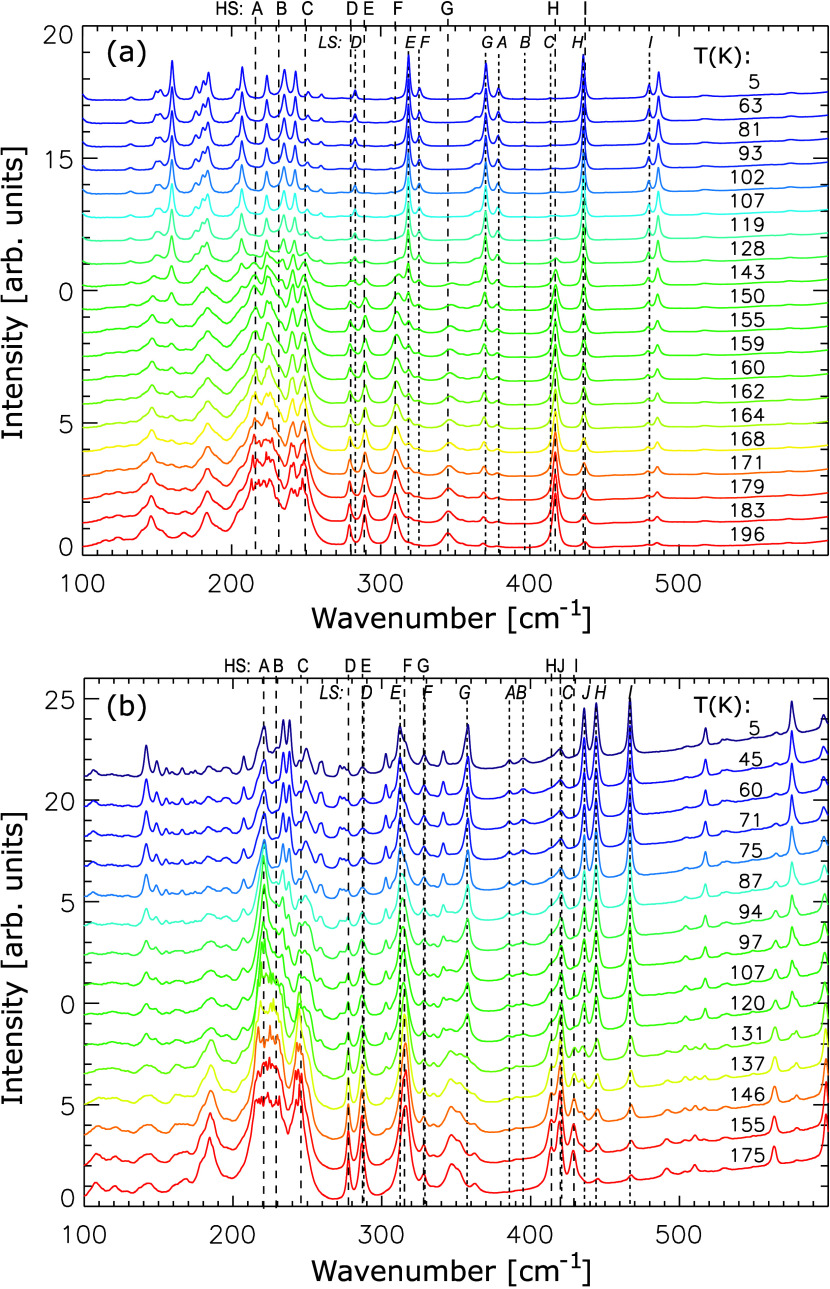
FTIR absorbance spectra (vertically stacked) in the FIR range for (a) mononuclear complex **1** and (b) dinuclear complex **2** as a function of temperature. Roman letters refer to HS, italic letters to LS vibrations.

Vibrational frequencies observed for monomer **1** and dimer **2** as well as calculated frequencies for the HS and LS configurations of these molecules are collected in Tables S1 and S2, respectively. With respect to the dimer, “LS” and “HS” refer to the LS-LS and HS-HS configurations, respectively; the mixed-spin HS-LS configuration will be analyzed later (see below). Nevertheless, correlations between HS and LS modes as well as between corresponding modes in the monomer and in the dimer can now be identified. Vibrational modes B (228 cm^–1^) and C (249 cm^–1^), e.g., correspond to antisymmetric Fe–N­(pz) stretching modes of the mononuclear complex **1** in the HS state at a high temperature (HT) of 196 K, shifting to 397 cm^–1^ (*B*) and 414 cm^–1^ (*C*) in the LS state at 5 K.^22^ Furthermore, the signal at 417 cm^–1^ (H) in the FIR spectrum of **1** at high temperature is due to the Fe–N symmetrical out-of-plane bend,^22^ shifting to 436 cm^–1^ in the LS state. For dinuclear complex **2**, the peaks at 229 (B) and 245 (C) cm^–1^ present in the HS state at 175 K shift to peaks at 395 (*B*) and 419 (*C*) cm^–1^ in the LS state at 5 K, in close similarity to the monomer. Notably, splitting of monomer bands into two bands in the dimer (corresponding to “gerade” (g) and “ungerade” (u) combinations of monomer vibrations) is not observed. We assume that this is due to the inversion symmetry of dimer **2**, rendering “g” combinations IR-forbidden. Other peak positions, assignments, and correlations between **1** and **2** can be inferred from Tables S1 and S2.

To investigate the influence of light illumination on the vibrational spectra of **1** and **2**, LIESST measurements were performed on both complexes at 5 K (Figure S3). Upon irradiation of monomer **1**, new bands associated with the HS state emerge on the background of the respective LS spectrum (Figure S3 Supporting Information; the fact that the intensity of the LS spectra remains constant with increasing irradiation time is a consequence of an incomplete excitation of the entire sample due to limited penetration depth of the radiation and the applied normalization procedure; see below). The spectrum obtained after completion of low-temperature irradiation (40 min), corresponding to the respective metastable HS state, is compared with the corresponding LS spectrum in Figure 4a.

**Figure 4:**
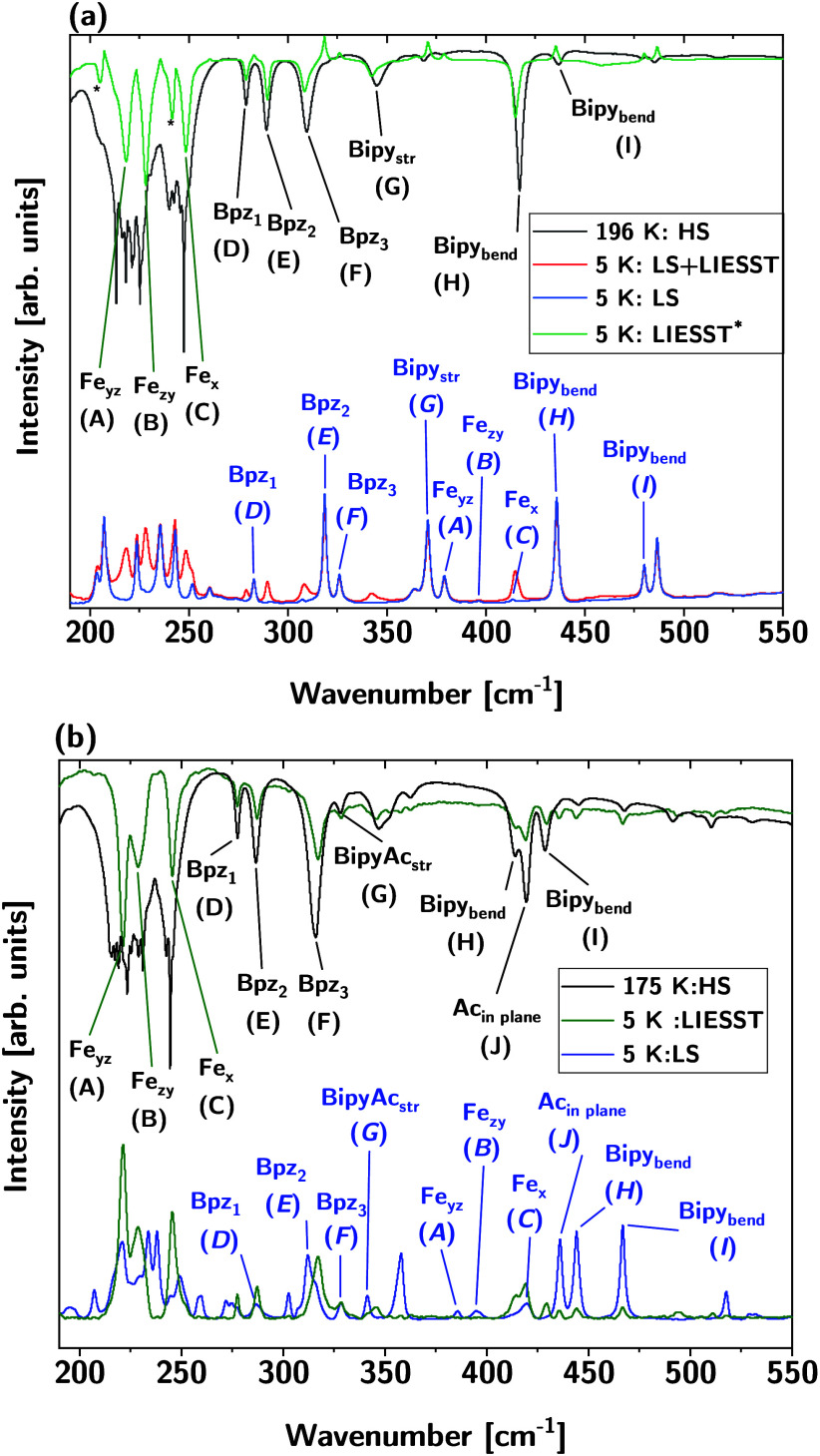
Assigned FIR spectra of (a) the mononuclear **1** and (b) the dinuclear complex **2** showing the spectra for high-temperature HS (black), 5 K LS (blue), and 5 K HS-LIESST (red for mononuclear, green for dinuclear). The LIESST spectrum of the mononuclear complex (green) is obtained by subtraction of the LS spectrum (blue) from the LS+LIESST (short illumination, red) spectrum. Peaks marked by an asterisk are not a feature of the measurement but a byproduct of the subtraction process. The LIESST spectrum of the dinuclear complex is obtained after 11 h of light exposure to receive complete SCO while for the mononuclear complex the LIESST spectrum was measured after 40 min of light exposure.

Figure 4a (bottom) shows the HS bands (red) emerging from the background of the LS spectrum (blue). The spectra are plotted with positive peaks to allow comparison with the high-temperature data (black), which are plotted in the conventional transmission mode (Figure 4a, top). For further comparison, the spectrum of the metastable HS state (green, LIESST) obtained by subtracting the low-temperature LS spectrum (blue) from the LS+LIESST spectrum (red) is plotted in Figure 4a, top, along with the (room temperature) HS spectrum. This presentation clearly shows the close correspondence between the spectrum of the metastable HS state (green) and the high-temperature HS state (black), although small frequency shifts are observable. Larger spectral differences can be noticed in the 200–250 cm^–1^ region, where the high-temperature data (black) are affected by noise. In this case, the green spectrum can be assumed to represent the “true” spectrum of the HS state.

Inspection of the data of Figure 4a shows that, after exposing the mononuclear complex **1** to light at low temperature, new bands appear in the low-frequency region at 218 (A), 228 (B), and 249 cm^–1^ (C), which correspond to Fe–N stretches of the HS state (see above).^22^ Note the large frequency shifts of bands A–C with respect to their counterparts in the LS spectrum which are located at 379, 397, and 414 cm^–1^, respectively. At higher frequencies, vibrations of the dihydrobis­(pyrazolyl)­borate (bpz) ligands (D–F) appear, which are also shifted with respect to the LS state. Moreover, a new bipy bending vibration is observed at 417 cm^–1^ (H) that also exhibits a considerable shift with respect to its LS counterpart located at 436 cm^–1^. Most conspicuously, the appearance of mode H at 417 cm^–1^ after LIESST indicates that the sample (or the part of it that has been subjected to illumination) has been converted to the metastable HS state. Such “marker” bands have been identified before to trace changes of the spin state in spin-crossover compounds.^43^

For dinuclear complex **2**, spectral data can be represented in an analogous way, allowing a similar analysis (Figure 4b). In monomer **1**, the presence of the LS modes after LIESST has been attributed to the fact that the penetration depth of the green light did not extend through the entire thickness of the pellet (see above and Figures S3 and S4). To account for this, the pellet of the dinuclear complex was illuminated with light from both sides for a longer time. In Figure 4b, bottom, the LIESST data obtained for the dinuclear complex after 11 h of light exposure at 5 K is plotted (green). Notably, a more complete conversion of the sample to the HS state compared with the monomer was achieved. This is evident from the fact that bands present in the LS spectrum (Figure 4b, bottom, blue) now appear with greatly reduced intensities in the LIESST spectrum (green).

In Figure 4b, top, the spectrum of the metastable HS state (green) is plotted along with the spectrum of the high-temperature HS state (black), showing the close correspondence between these spectra. Again, bands in the low-frequency region are associated with Fe–N stretches, i.e., at 221 (A), 229 (B), and 245 cm^–1^ (C). Interestingly, the dinuclear complex shows an intense peak at 186 cm^–1^ after LIESST (Figure S3), which corresponds to a combined vibration of the bpz- and bipyacbipy-ligands and is therefore not observed for the mononuclear complex. At higher frequencies, vibrations of the bpz ligands (D, E, and F) appear, which are also shifted with respect to the LS state. Moreover, new bipy bending vibrations are observed at 417 cm^–1^ (H) and 429 cm^–1^ (I), which also exhibit considerable shifts with respect to their LS counterparts. Note the “splitting” of the 417 cm^–1^ band (H) not being present in the monomer data. Due to the inversion symmetry of dimer **2**, a “g/u” splitting can be excluded to account for this observation (see above). Based on DFT we assign the second component (J) to an Ac­(inplane) vibration involving the acetylene (Ac) unit contained in the bridging ligand (cf. SI Figure S2).

Analysis of the LIESST data thus has provided complementary information to the temperature-dependent measurements and thus helped to confirm the spectral assignments as well as the associated shifts (Tables S1 and S2). Specifically, the vibrational modes of the iron center along the axes of the FeN_6_ octahedron (A–C) shift upon SCO from HS (200–250 cm^–1^) to LS (370–420 cm^–1^).^44,45^ In addition to the Fe–N stretching modes, spin-sensitive vibrational modes of both the pyrazolborate ligands (bpz) and the (bridging) bipyridine ligand(s) (bipy/bipyacbipy) have been identified. These ligand modes can be found between 270 and 320 cm^–1^ for bpz and >420 cm^–1^ for bipy and bipyacbipy. The shifts of these vibrations range between 5 and 70 cm^–1^ and are therefore considerably smaller than those of the iron center. Nevertheless, they also can be traced well, which leads to valuable information regarding the vibrational structures of **1** and **2** in their respective spin states.

To obtain information about the nature of the spin transition process, the temperature-dependent intensities of the spin-sensitive vibrational modes were analyzed. All spectra obtained at different temperatures were corrected to a baseline unique for each peak position; an example is shown in Figure S5. The intensities of selected high- and low-temperature modes are plotted against the temperature in Figure 5a for **1** and in Figure 5b,c for **2**.

**Figure 5:**
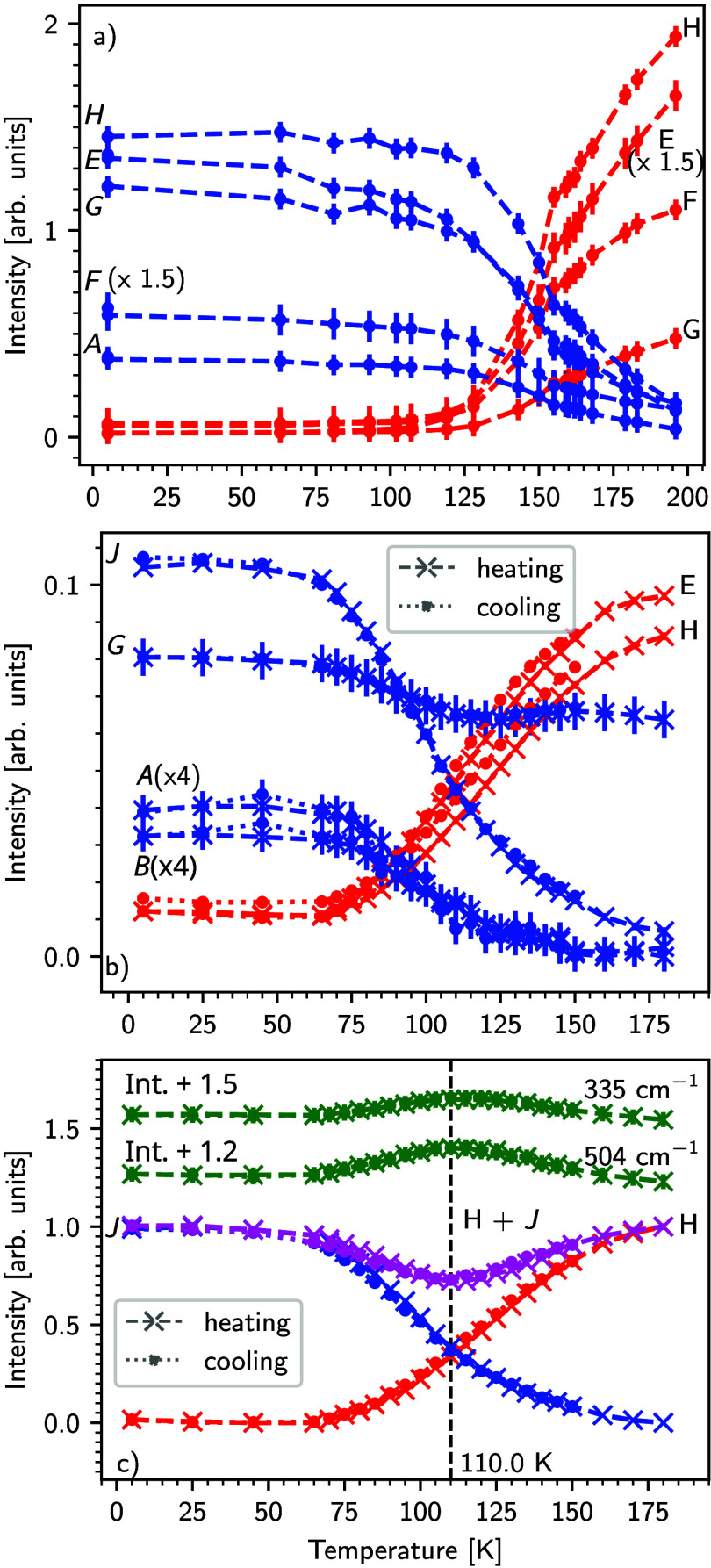
(a) Decay and rise of some assigned peaks according to Table S1 vs temperature for the spectra from Figure 3 for mononuclear complex **1**. The cooling rate was maintained at 1–4 K/min below *T*_1/2_ until 80 K [cooling rates for the whole temperature ranges are shown in Figure S6]. (b) Evolution of the peak intensity for selected wavenumbers for heating and cooling cycles (1 K min^–1^) of the dinuclear complex (**2**). (c) Sum of HS-HS (H) and LS-LS (*J*) peaks after normalization, which is inverse to the behavior of the 335 cm^–1^ and 504 cm^–1^ peaks.

The denominations of the modes correspond to Figures 3, S1, and S2 as well as Tables S1 and S2. The absorbance vs temperature curves for different high- and low-temperature modes cross at ≈ 150 K for the mononuclear complex and at ≈ 110 K for the dinuclear complex, at the respective *T*_1/2_ values obtained from SQUID and XAS measurements (cf Figure 2).^5,22,23^

Interestingly, in the intermediate temperature range 94 K–120 K, in Figure 3 distinctive signals at 335 and 504 cm^–1^ appear, which arise upon the start of the spin transition, peak in intensity approximately at *T*_1/2_, persist in the intermediate temperature range, and then decrease in intensity after completion of the spin transition. Vibrational modes with such a temperature-dependent intensity profile could be related to a mixed spin state (HS-LS) of **2** as they are not observed at high and low temperatures where, due to the complete SCO behavior of the dinuclear complex, only the HS-HS and, respectively, LS-LS states exist.

In order to obtain further insight into the origin of the 335 and 504 cm^–1^ peaks, the sum of a selected HS-HS (H, 414 cm^–1^) and a LS-LS (*J*, 436 cm^–1^) mode is plotted in Figure 5 b after normalization. The intensity profile of this sum is inverse to those of the 335 and 504 cm^–1^ peaks, indicating that the 335 and 504 cm^–1^ peaks must be related to the HS-LS state. To rule out contributions from the HDPE within this temperature range, we have measured HDPE at different temperatures (Figure S7). The peak intensities of HDPE did not show a temperature dependence in the 504 cm^–1^ region (Figure S7b).

In principle, vibrational modes that are exclusive to the HS-LS state must originate from vibrations of the bridging ligand. This is because the two iron centers of the HS-LS system are only weakly coupled. Therefore, as evident from Table S4, Fe–N stretching modes as well as vibrations of the surrounding ligands of this system appear twice. In one component, primarily the HS center vibrates (with weak contributions from the LS center), and the other component primarily involves the LS center (with small contributions from the HS center). The frequencies of these more or less “local” vibrations roughly correspond to those observed for the HS-HS and LS-LS states, respectively. Consequently, if the HS-LS state is traced by the presented method, one has to look for additional bands which are absent in both HS-HS and LS-LS complexes. Such signals arise in regions in which vibrational modes of the bridging ligand are found.

As described above, the DFT-analysis provided insight about the spectral region in which vibrational modes of the bridging ligands are located or even found exclusively, which depends on the spin state. These modes are typically found between 360 and 600 cm^–1^ for the HS-HS state. For the LS-LS state, the lower limit of this region shifts significantly to higher wavenumbers, while the upper limit stays about the same (435–600 cm^–1^). In this region, e.g., the additional peak observed at 504 cm^–1^ for the HS-LS state is located (see above). It can be assigned to a vibrational mode composed of an asymmetric twisting motion of the bipyridine units combined with an in-plane vibration of the acetylene unit (Figure 6 and Table S4).

**Figure 6:**
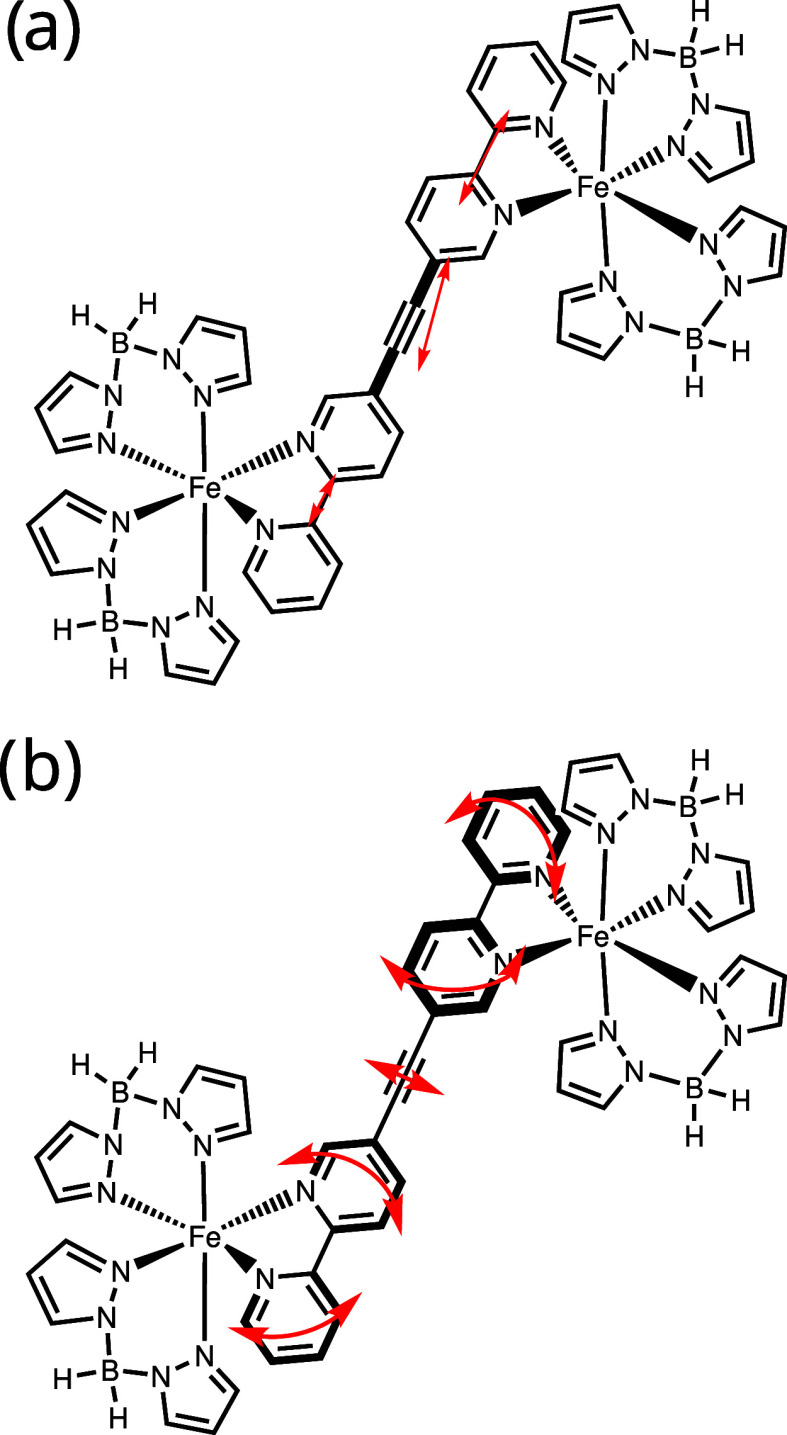
Vectorial depiction of specific vibrational modes of the HS-LS-state of the dinuclear complex (**2**) (a) 335 cm^–1^: BipyAc*_str_* (mode G) and (b) 504 cm^–1^: Bipyacbipy-twisting combined with an in-plane acetylene vibration.

At 339 cm^–1^, a stretching mode of the bridging ligand (BipyAc stretch) is calculated which is assigned to the signal observed at 335 cm^–1^ (cf. Table S4); i.e., between the frequencies of 328 cm^–1^ observed for the HS-HS (G) and 341 cm^–1^ observed for the LS-LS (*G*) state. The frequency increase is due to the increasing strength of the bonds within the bridging ligand upon going from HS to LS. The stronger Fe–N bonds in the LS-LS state also lead to combined vibrations of the bridging ligand and the Fe centers, whereas in the HS-HS case motions of the bridging ligand are decoupled from the latter; i.e., they occur as independent vibrations. The mixed HS-LS vibration represents a combination of both cases, which is also visible in the pictorial representation of its eigenvector (cf. Figure 6). Thus, contributions from both bipyridine units to the HS and LS centers are visible.

In summary, the emergence of the described bands indicates the presence of a mixed-spin LS-HS state of dimer **2** that is populated in the vicinity of *T*_1/2_ in the course of the thermal spin transition from HS-HS to LS-LS and vice versa.

To explore the switching transition dynamics under irradiation with light, rate constants were determined by applying an exponential fit (*I* = *k*_1_ + *k*_2_·exp­(−*t*·*r*)) to the intensity change of different bands over time (Figure 7 and Figure S8), where *t* is the laser exposure time, *r* is the rate constant for LIESST, *k*_1_ is the fraction of complexes that does not switch with light, and *k*_2_ is the fraction of complexes that react to light. Notably, there are slight differences in the vibrational modes of **1** and **2** [Table S3]. The fact that not all modes exhibit the same rate constants indicates that different parts of the molecule adapt in slightly different ways to the geometrical changes involved in the transition from the LS to the HS state (Figure 7 and Figure S8). This provides information in a mode-selective way; i.e., at submolecular resolution, regarding the (anisotropic) structural dynamics of the individual molecule occurring upon asymmetric change of the spin-state. In particular, it seems that the Fe–N asymmetric stretching modes of the dinuclear complex at 221 cm^–1^ (A), 229 cm^–1^ (B), and 245 cm^–1^ (C) show a quick intensity change with light [faster than other modes like 414 cm^–1^ (H)], as these modes are most affected by the spin-state transition (Figure 7, Table S3). Furthermore, the time-dependent IR spectra of dimer **2** do not show the appearance of any new peak over time (Figure S4). In particular, the peaks at 335 and 504 cm^–1^ are not observed; i.e., the data only reflect intensity changes of HS-HS and LS-LS modes (Figure S4). These results indicate that the light-induced spin transition in the dimer occurs more or less directly from LS-LS to HS-HS. This agrees with the fact that the thermal spin transition of this system proceeds in a single step.^23^ Notably, a two-step transition is observed if the transition of one center from HS to LS (coming from RT) distorts the other (HS) center such that its ligand field strength decreases.^46–48^ However, the Fe–N distances calculated for the HS-LS states of 2 agree with those calculated for the HS-HS and LS-LS states (cf., Table S5), respectively, excluding such an effect. The same conclusion can be drawn from the frequencies of the Fe–N stretches, which for the HS-LS state are very similar to those found for the HS-HS and LS-LS states, respectively (see above). Finally, an energetic criterion for the occurrence of a stepwise spin-transition in a dinuclear complex is that the enthalpy of the HS-LS state is lower than the midpoint (**M**) between the enthalpies of the LS-LS and HS-HS states.^49^ However, we find (see Table S6) that the enthalpy/energy of the HS-LS state within 0.1 kJ/mol (i.e., almost exactly) corresponds to **M**. Of course, the surrounding lattice may also influence the energy of the HS-LS state.^49^ The observation that the fraction of the HS-LS state around *T*_1/2_ is low suggests that the energy of this state is in fact slightly above **M**,^50^ which in turn might be due to a bad fit of the asymmetric HS-LS molecule into the lattice.^51^ In any case, this situation would be compatible with the thermally and light-induced spin-state switching of **2** occurring in one step.

**Figure 7:**
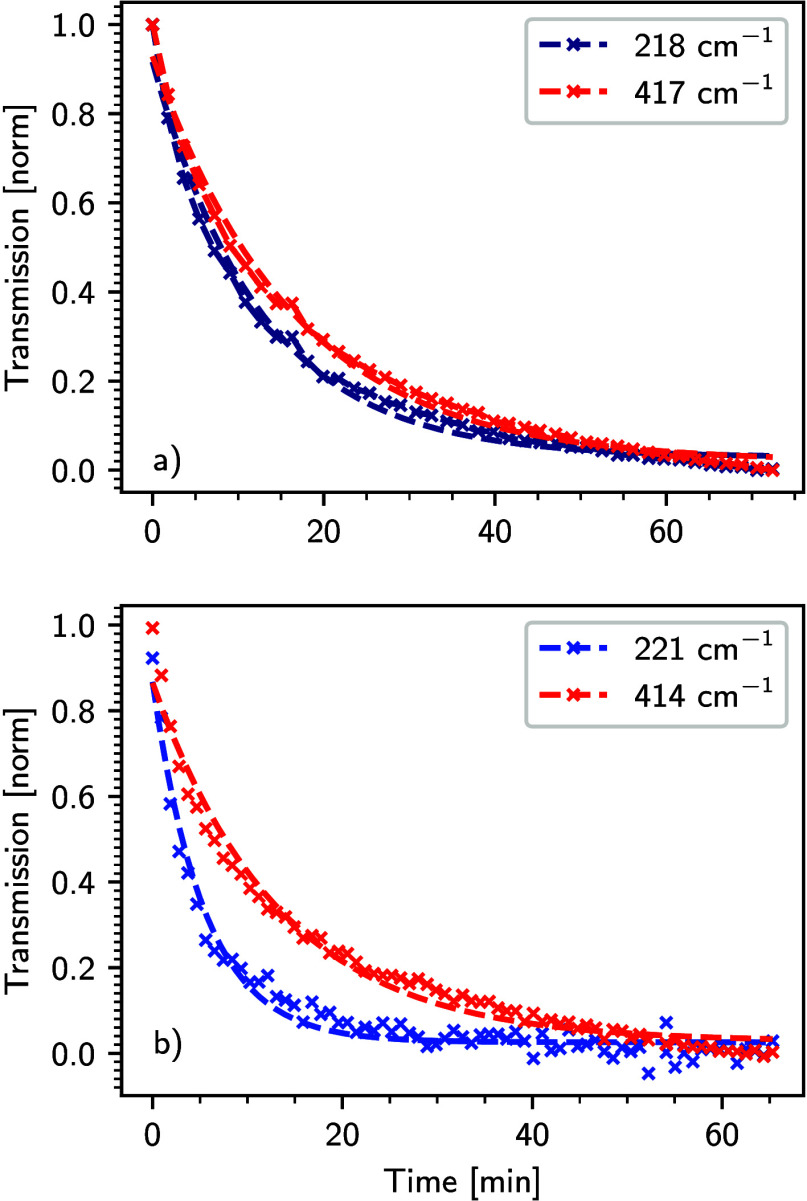
Intensity change of modes A [218 (221) cm^–1^] and H [417 (414) cm^–1^] after switching on the light as a function of time at 5 K in (a) the mononuclear complex (**1**) and (b) the dinuclear complex (**2**). Dotted lines show the exponential fits to the normalized transmission intensity. The obtained kinetics are given in Table S3, Supporting Information for the mononuclear (**1**) and the dinuclear complex (**2**), respectively.

Finally, the LIESST experiments indicate that the excitation to the metastable HS complex is faster for the dimer than for the monomer for two metal–ligand stretching modes. An exact understanding of this observation is difficult at the present stage. It should be noted that earlier measurements on the dinuclear complex^23,32^ did not show much influence of the nuclearity of the spin-crossover complex on the kinetics of the LIESST effect due to the pronounced effect of soft-X-ray-induced excited spin-state trapping but already indicated that the dinuclear complex can be switched by light, in agreement with UV/vis absorption experiments.^23^

Whereas the present study has mostly focused on the FIR range, both complexes were also investigated in the MIR region as a function of temperature and light (Figures S9, S10, S11, S12, and S13). The data shown in the SI indicate the presence of vibrational shifts between the spin states of the mononuclear as well as the dinuclear complex, similar to the FIR region. These data can be analyzed in a similar fashion as performed for the FIR range, providing complementary information.

A comparative FIR study of a mononuclear and a dinuclear Fe (II) complex indicates that the dinuclear complex undergoes thermal spin crossover from HS-HS to LS-LS essentially in a single step, showing a weak population of the HS-LS state around *T*_1/2_. This is inferred from characteristic vibrational modes that arise at 335 cm^–1^ and 504 cm^–1^ during the spin transition temperature and peak at *T*_1/2_. Supported by DFT, the detailed analysis of the vibrational modes indicates that the two iron centers within the dinuclear complex exhibit only weak coupling. The LIESST measurements reveal that the dinuclear complex reacts to light by also following a direct spin-state transition from LS-LS to HS-HS. Around *T*_1/2_ the number of complexes in the HS-LS configuration is small, which presumably is a consequence of the lack of energetic stabilization of this state. These results underscore that temperature-dependent IR measurements are a powerful tool to trace and detect details of the spin-crossover process that are difficult to access conveniently by already-established methods, thus affording a valuable complementary method.

## Supplementary Material



## Notes

Sample preparation and measurement, density functional theory, baseline correction, MIR measurements, vectorial depiction and experimental data and calculations of the vibrational modes of the mononuclear complexes **1** and **2**, LIESST excitation rates, and additional spectra and data (PDF)
