# Supplementary material for: Tracking the HS-HS, HS-LS, and LS-LS States in the Spin Transition of a Dinuclear Fe(II) Complex by Broadband FTIR Spectroscopy
Source: J Phys Chem Lett. 2025 Dec 5;16(50):12675–83. doi: 10.1021/acs.jpclett.5c03119 (PMC12720236; doi:10.1021/acs.jpclett.5c03119)
Supplement: Supplementary file 1 [file jz5c03119_si_001.pdf]

Tracking the HS-HS, HS-LS and LS-LS states in the spin transition of a dinuclear Fe(II) complex by broadband FTIR spectroscopy

Marcel Walter,[†,§] Eike F. Kuhlemann,[‡,§] Tarek Al Said,[¶] Clara W.A. Trommer,[‡] Felix Tuczek,[*,‡] Karsten Holldack,[*,¶] Wolfgang Kuch,[*,†] and Sangeeta Thakur[*,†]

†*Institut für Experimentalphysik, Freie Universität Berlin, Arnimallee 14, 14195 Berlin, Germany*

‡*Institut für Anorganische Chemie, Christian-Albrechts Universität zu Kiel, 24098 Kiel, Germany*

¶*Helmholtz-Zentrum Berlin für Materialien und Energie GmbH, Hahn-Meitner-Platz 1, 14109 Berlin, Germany*

§*These authors contributed equally to this work*

E-mail: ftuczek@ac.uni-kiel.de; karsten.holldack@helmholtz-berlin.de; kuch@physik.fu-berlin.de; sangeeta.thakur@fu-berlin.de

Supplementary Information

Sample preparation and measurement

The vibrational properties of the Fe(II) bulk complexes were measured by an optimized FTIR setup for both, the FIR and MIR range with a Bruker IFS125 HR spectrometer as a function of temperature (5 to 200 K) and light exposure (520 nm) at the THz beamline at BESSY II. The employed broadband Si bololmeter detector enabled very-far FIR (THz) to

MIR measurements and provides good intensity also in the low < 600 cm^{-1} region. For the FTIR measurements, samples were prepared as pellets (10 mm diameter, $\approx$ 2 mm thickness, $\approx$ 1000 psi) from a mix of the complex powder with high-density polyethylene (HDPE) for FIR measurements or with KBr for MIR measurements. For FIR measurements, the spectrometer was equipped with an Hg lamp, a 6 µm thick Mylar beamsplitter and a liquid-helium-cooled bolometer detector. For MIR measurements, a globar, **MCT detector** and a KBr beamsplitter were used. Spectra were taken at a resolution of 1 and 2 cm^{-1}. To investigate the kinetics of the spin transition in the dinuclear complex, measurements were done at heating and cooling rates of 1 K/min. In this case, the spectra were measured after the temperature stabilized, which was achieved by waiting for approximately 15 minutes. Light excitation was performed with a green laser diode (532 nm, spot size < 10 mm, power $= 10$ mW), which was coupled in through quartz windows into the evacuated spectrometer. FIR spectra were accumulated during light illumination at 5 K, and after switching off the light, the final saturated spectrum was measured. To maximize the fraction of molecules in the metastable HS state after LIESST, the mononuclear and dinuclear complexes, were illuminated with green light for 40 and 80 min., respectively. In another experiment, the dinuclear complex was exposed to light for 11 hrs. To obtain transmittance (T) spectra of the sample only, all spectra are divided by a reference HDPE spectrum. To observe also small changes, absorbance spectra are plotted as [log(1/T)].

Density Functional Theory (DFT)

For the analysis of the IR data, DFT was employed to assign the signals of the spectra to vibrational modes of the compounds. Tables S1 and S2 show the wavenumber of the modes observed experimentally and obtained by DFT calculations using Gaussian16 with a uB3LYP-def functional with a 6-311G basis set for first- and second-row atoms and lan12dz for Fe or Orca 4.2.1 with the functionals BP86, PBE, PBE0, B3LYP and OLYP with def2svp or def2tzvp basis sets. The mixed-spin state (HS-LS) of the dimer was examined using Orca 6.1 with a TPSSh def2-tzvp functional-basis set combination. The mixed-spin state was

converged to a satisfying solution by first converging the structure of a Zn-Fe dimer with an appropiate geometry for a HS-LS dimer and the following resubstitution of Zn for Fe. The resulting peak assignment of spin-sensitive modes in the range of 200-550 cm^{-1} is shown in Figure 4. As expected, all DFT calculations show that the vibrational modes of the iron center along the axis of the coordination polyeder (A-C) are shifting upon SCO from HS (200-250 cm^{-1}) to LS (370-420 cm^{-1}). In order to assign the peaks in the measured spectra based on the data provided by DFT, the IR-active modes of the HS- and LS- calculation were animated to identify the corresponding modes for each spin state. The resulting shifts were compared throughout all applied methods and the peaks of the measured spectra were assigned accordingly. To minimize the potential for mistaken assignments, the peaks were reassigned until substantive agreement was achieved between the measured spectra and the calculated data. The HS, LS, and LIESST spectra of both the mononuclear and dinuclear complex are shown in Figure 4, with assigned peaks referencing the substructure of the complex ($Fe_{x,y,z}$, *Bpz*, *Bipy*). Peak position and shifts upon SCO are given in Tables S1 and S2. For the LIESST spectra at 5 K, in addition to the HS modes, also LS modes are present for both complexes (Table S2, S1). For the identification of spin-sensitive peaks in the range of 200-250 cm^{-1} in the HS spectrum, the 5 K HS-LIESST spectra were used exclusively to avoid systematic errors owing to some obvious saturation effect in the high-temperature HS spectra, rendering this particular fraction of the high-temperature HS spectrum unsuitable for evaluation. When comparing the spectra of the mononuclear (**1**) and the dinuclear (**2**) complexes, the basic peak structure noticeably is, with small deviations, the same for both complexes. This can be expected as the coordination motive of **1** is basically reproduced in **2** and extended by the addition of an acetylen bridge. Even as this brigde adds vibrational modes to the spectra around 415 cm^{-1} by coupling the bipyridine-ligands, it does not substantially change the overall shifting behaviour. Consequently, the two iron sites in **2** can be assessed as weakly coupled.

Baseline Correction

Absorbance in absolute terms means a spectrum from the sample (µm-sized grains of a few mg pressed into 100 mg HDPE) divided by the spectrum of a 100 mg HDPE reference sample. For each measurement, a reference pellet of 100 mg HDPE was made without SCO molecule. All pellets are pressed with $\approx$ 1000 psi resulting in a a disk of 10 mm in diameter. Absorbance is calculated as A=log (1/Transmittance(T)), while T is T=spectrum-sample/spectrum-HDPE measured under the same detection conditions. For further analysis, the relative absorbance between peaks at different temperatures was compared by calculating their area after baseline correction. We also measured the temperature dependence of the empty HDPE reference (Fig. S7), to check for background artifacts arise due to temperature-dependent bands in the HDPE next to the peaks of interest. This was carefully done by subtracting the background directly next to the peaks (Fig. S5) to remove systematic errors from the temperature-dependent distortion of the overall FTIR spectrum envelope. This distortion is mainly caused by (tiny) thermal distortion of the low-temperature sample compartment inside the magnet.

MIR-measurements

For **1**, the bands at 717 and 751 cm^{-1} arise due to the HS state at 200 K, while for the LS state, an intense band appears at 746 cm^{-1} and 764 cm^{-1} at 5 K.[1] From the comparison of the MIR spectra of **2** with **1**, we can conclude that the bands at 750 cm^{-1} and 747 cm^{-1} are characteristic of the HS and the LS state, respectively. For both complexes, the appearance of the intense band around 750 cm^{-1} and the disappearance of the band at 747 cm^{-1} after LIESST means that the complex is in the metastable HS state after LIESST. Some of the bands which are characteristic of the LS state are also present after LIESST, as observed for the FIR region. The LIESST measurements were done on **1** and **2** for 288 and 80 min., respectively. Figure S13 shows changes in position and intensity of the peaks of **2** with time during LIESST. The spin-state transition for **2** under light illumination is fast in comparison to **1**.

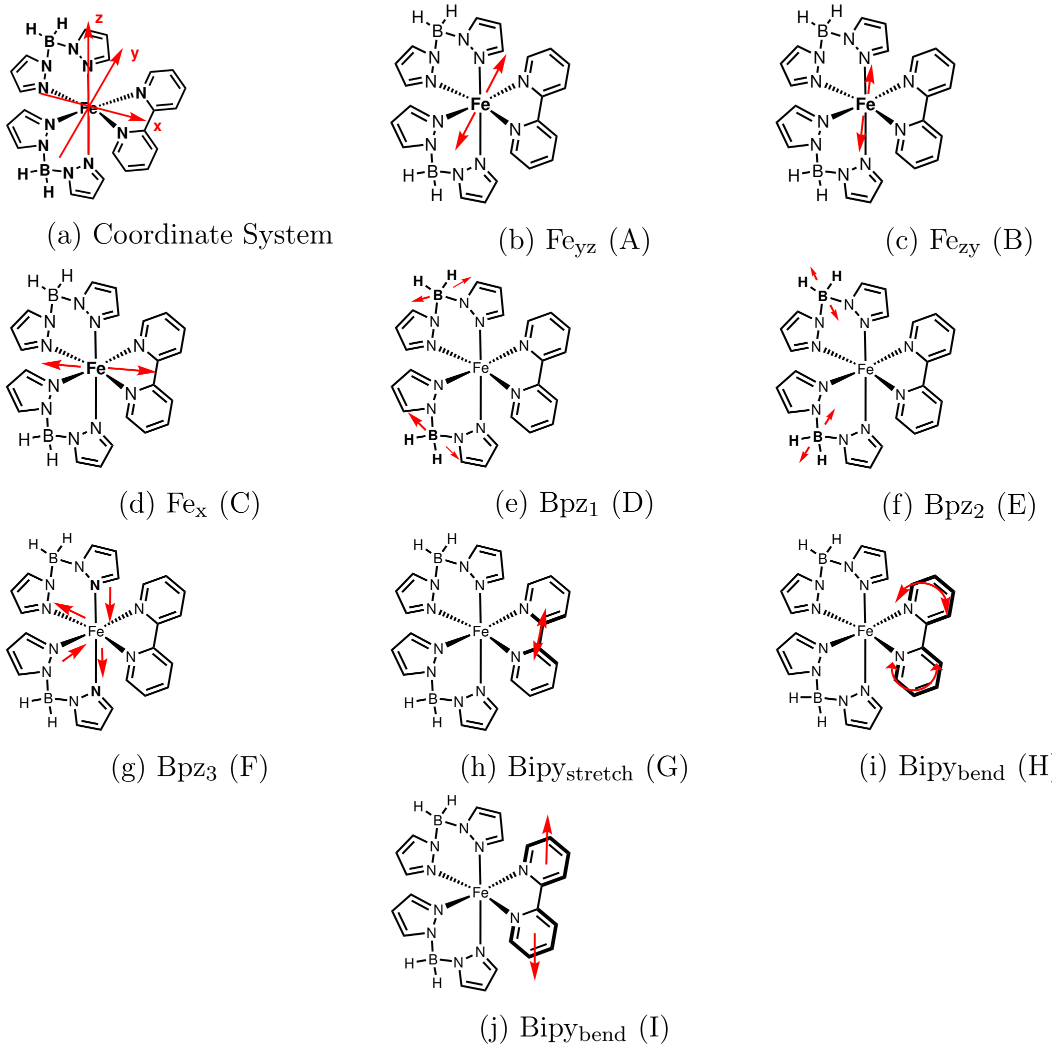

(a) Coordinate System (b) Fe_{yz} (A) (c) Fe_{zy} (B)

(d) Fe_{x} (C) (e) Bpz_{1} (D) (f) Bpz_{2} (E)

(g) Bpz_{3} (F) (h) $Bipy_{stretch}$ (G) (i) $Bipy_{bend}$ (H)

(j) $Bipy_{bend}$ (I)

Figure S1: Vectorial depiction of the vibrational modes of the mononuclear complex (**1**).

Table S1: Vibrational modes of the mononuclear complex (**1**): experimental data and calculations (a) BP86-def2svp, b) PBE0-def2svp, c) B3LYP-def2tzvp, d) uB3LYP-6-311G, lan12dz).

	HS/LIESST cm^{-1}		LS cm^{-1}		Shifts (LS-HS) cm^{-1}	
Mode	assigned ($\pm$ 0.2 cm^{-1})	calculated	assigned ($\pm$ 0.2 cm^{-1})	calculated	assigned ($\pm$ 0.2 cm^{-1})	calculated
Fe_{yz} (A)	218	a) 273<br>b) 237<br>c) 226<br>d) 239	379	a) 426<br>b) 394<br>c) 397<br>d) 395	161	a) 153<br>b) 157<br>c) 171<br>d) 156
Fe_{zy} (B)	228	a) 232<br>b) 247<br>c) 240<br>d) 199	397	a) 392<br>b) 403<br>c) 385<br>d) 385	169	a) 159<br>b) 156<br>c) 145<br>d) 186
Fe_{x} (C)	249	a) 223<br>b) 252<br>c) 237<br>d) 274	414	a) 414<br>b) 422<br>c) 400<br>d) 400	172	a) 191<br>b) 170<br>c) 163<br>d) 126
Bpz_1 (D)	279	a) 322<br>b) 321<br>c) 320<br>d) 318	283	a) 324<br>b) 326<br>c) 318<br>d) 315	4	a) 2<br>b) 5<br>c) -2<br>d) -3
Bpz_2 (E)	289	a) 285<br>b) 287<br>c) 286<br>d) 283	319	a) 319<br>b) 319<br>c) 315<br>d) 313	30	a) 34<br>b) 32<br>c) 29<br>d) 30
Bpz_3 (F)	310	a) 318<br>b) 298<br>c) 323<br>d) 316	326	a) 361<br>b) 309<br>c) 366<br>d) 361	16	a) 43<br>b) 11<br>c) 43<br>d) 45
$Bipy_{str}$ (G)	345	a) 354<br>b) 357<br>c) 353<br>d) 356	371	a) 376<br>b) 374<br>c) 371<br>d) 379	27	a) 22<br>b) 17<br>c) 18<br>d) 14
$Bipy_{bend}$ (H)	417	a) 422<br>b) 426<br>c) 429<br>d) 424	436	a) 434<br>b) 440<br>c) 453<br>d) 456	19	a) 12<br>b) 14<br>c) 24<br>d) 32
$Bipy_{bend}$ (I)	437	a) 435<br>b) 452<br>c) 444<br>d) 454	480	a) 464<br>b) 473<br>c) 466<br>d) 477	43	a) 29<br>b) 20<br>c) 22<br>d) 23

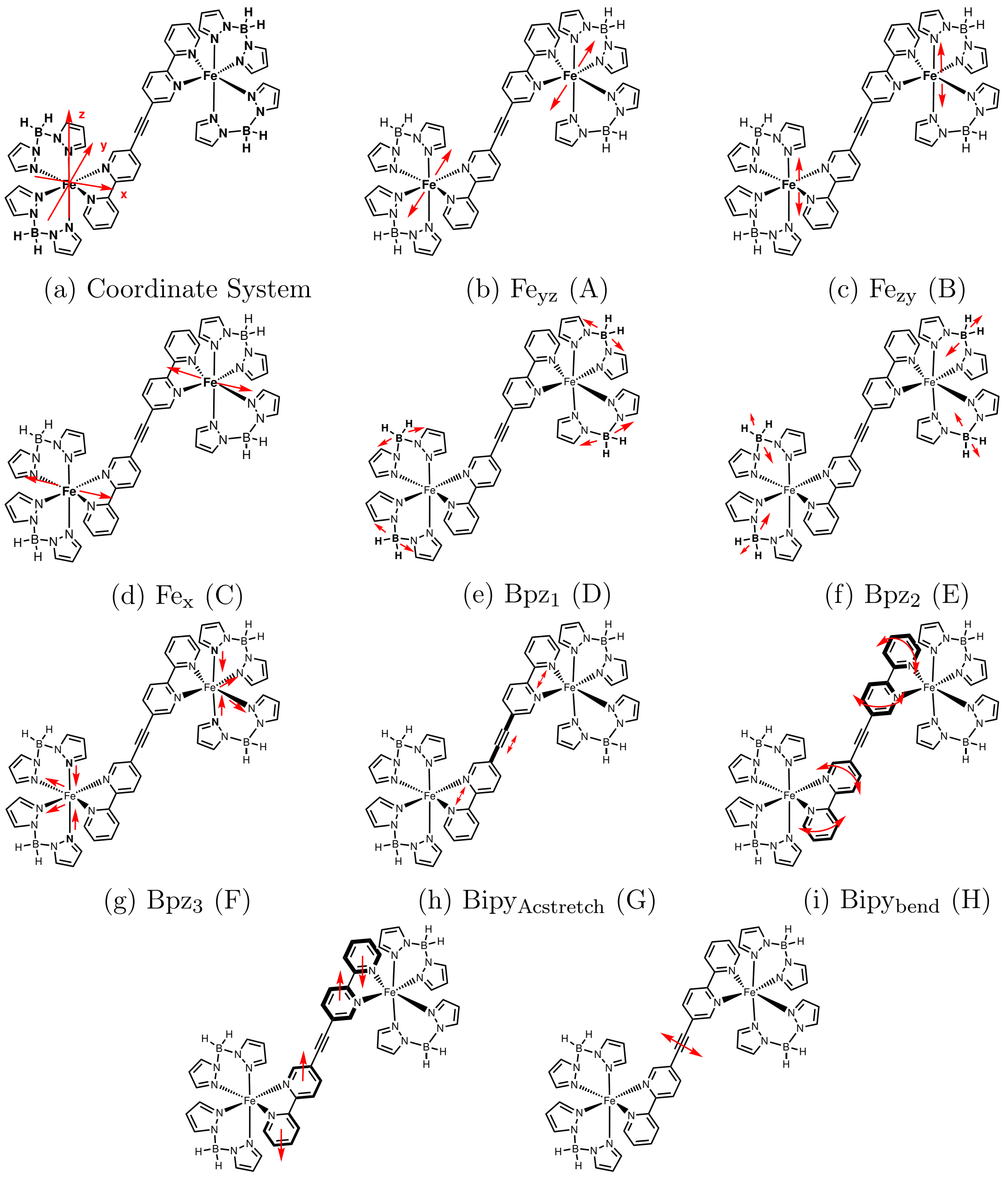

(a) Coordinate System (b) $\mathrm{Fe_{yz}}$ (A) (c) $\mathrm{Fe_{zy}}$ (B)

(d) $\mathrm{Fe_{x}}$ (C) (e) $\mathrm{Bpz_1}$ (D) (f) $\mathrm{Bpz_2}$ (E)

(g) $\mathrm{Bpz_3}$ (F) (h) $\mathrm{Bipy_{Acstretch}}$ (G) (i) $\mathrm{Bipy_{bend}}$ (H)

(j) $\mathrm{Bipy_{bend}}$ (I) (k) $\mathrm{Bipy_{Ac\ in\ plane}}$ (J)

Figure S2: Vectorial depiction of the vibrational modes of the dinuclear complex (**2**).

Table S2: Vibrational modes of the dinuclear complex (**2**): experimental data and calculations (a) BP86-def2tzvp, b) PBE-def2tzvp, c) OLYP-def2tzvp, d) TPSSh-def2tzvp).

	HS/LIESST cm^{-1}		LS cm^{-1}		Shifts (LS-HS) cm^{-1}	
Mode	assigned ($\pm$ 0.2 cm^{-1})	calculated	assigned ($\pm$ 0.2 cm^{-1})	calculated	assigned ($\pm$ 0.2 cm^{-1})	calculated
Fe_{yz} (A)	221	a) 266<br>b) 247<br>c) 239<br>d) 232	386	a) 417<br>b) 411<br>c) 413<br>d) 410	165	a) 188<br>b) 164<br>c) 174<br>d) 178
Fe_{zy} (B)	229	a) 238<br>b) 222<br>c) 220<br>d) 221	395	a) 391<br>b) 388<br>c) 394<br>d) 395	166	a) 153<br>b) 166<br>c) 174<br>d) 174
Fe_{x} (C)	245	a) 222<br>b) 232<br>c) 268<br>d) 261	419	a) 410<br>b) 404<br>c) 422<br>d) 413	175	a) 188<br>b) 172<br>c) 154<br>d) 152
Bpz_1 (D)	278	a) 280<br>b) 279<br>c) 283<br>d) 276	287	a) 276<br>b) 292<br>c) 279<br>d) 292	9	a) -4<br>b) 13<br>c) -4<br>d) 16
Bpz_2 (E)	287	a) 284<br>b) 284<br>c) 285<br>d) 280	312	a) 315<br>b) 311<br>c) 310<br>d) 309	25	a) 31<br>b) 27<br>c) 25<br>d) 29
Bpz_3 (F)	315	a) 314<br>b) 301<br>c) 315<br>d) 315	328	a) 327<br>b) 323<br>c) 322<br>d) 327	13	a) 13<br>b) 22<br>c) 7<br>d) 12
$BipyAc_{str}$ (G)	328	a) 343<br>b) 325<br>c) 335<br>d) 331	341	a) 353<br>b) 344<br>c) 352<br>d) 346	13	a) 10<br>b) 19<br>c) 17<br>d) 15
$Bipy_{bend}$ (H)	414	a) 415<br>b) 416<br>c) 424<br>d) 419	444	a) 445<br>b) 444<br>c) 448<br>d) 438	30	a) 30<br>b) 28<br>c) 24<br>d) 19
$Bipy_{bend}$ (I)	429	a) 423<br>b) 436<br>c) 445<br>d) 431	467	a) 457<br>b) 456<br>c) 459<br>d) 459	38	a) 34<br>b) 20<br>c) 14<br>d) 28
$Ac_{in\ plane}$ (J)	420	a) 420<br>b) 421<br>c) 428<br>d) 425	436	a) 440<br>b) 438<br>c) 446<br>d) 444	16	a) 20<br>b) 17<br>c) 22<br>d) 19

Table S3: LIESST excitation rates of the mononuclear (**1**) and dinuclear (**2**) complex

band (cm^{-1})	$r \times 10^{-2}$ (min^{-1})	$k_1 \times 10^{-2}$	k_2
218 (A) (**1**)	7.9(3)	2.9(7)	0.89(2)
228 (B) (**1**)	6.1(2)	2.2(8)	0.90(2)
249 (C) (**1**)	7.5(3)	2.7(7)	0.89(2)
417 (H) (**1**)	6.1(2)	1.9(7)	0.91(1)
186 (**2**)	7.1(3)	3.4(7)	0.87(1)
221 (A) (**2**)	18.4(8)	7.3(5)	0.84(2)
229 (B) (**2**)	11.0(5)	5.2(6)	0.83(2)
245 (C) (**2**)	7.6(3)	3.7(7)	0.84(2)
287 (E) (**2**)	7.6(3)	3.3(7)	0.86(2)
315 (F) (**2**)	8.4(3)	4.5(7)	0.84(2)
414 (H) (**2**)	7.5(3)	3.4(7)	0.84(2)

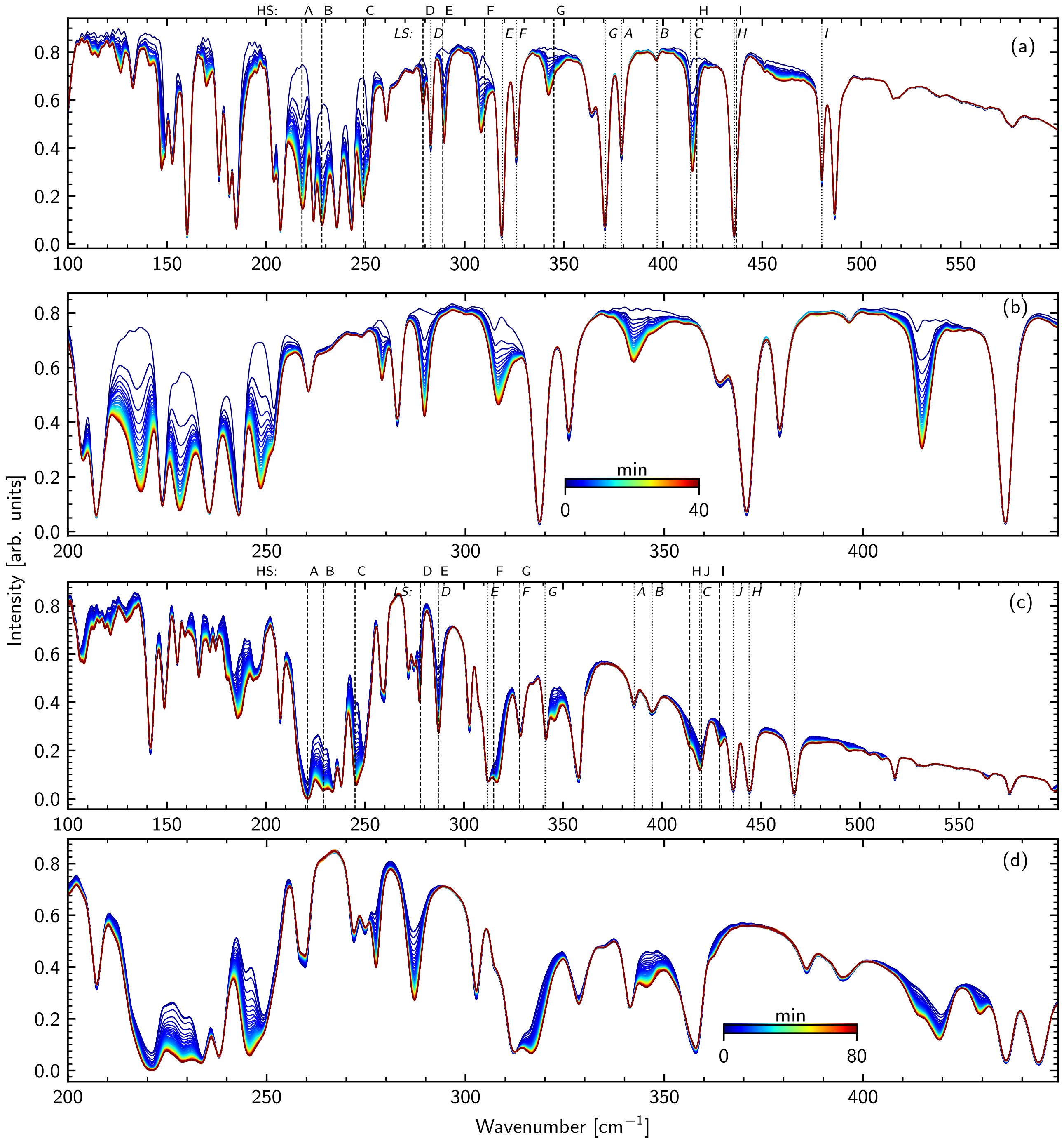

Figure S3: FTIR transmittance spectra of (a) the mononuclear complex (**1**) (c) dinuclear complex (**2**) color-coded as a function of exposure time with a 520 nm green laser, with corresponding closeups in (b) and (d) respectively, measured at 5 K.

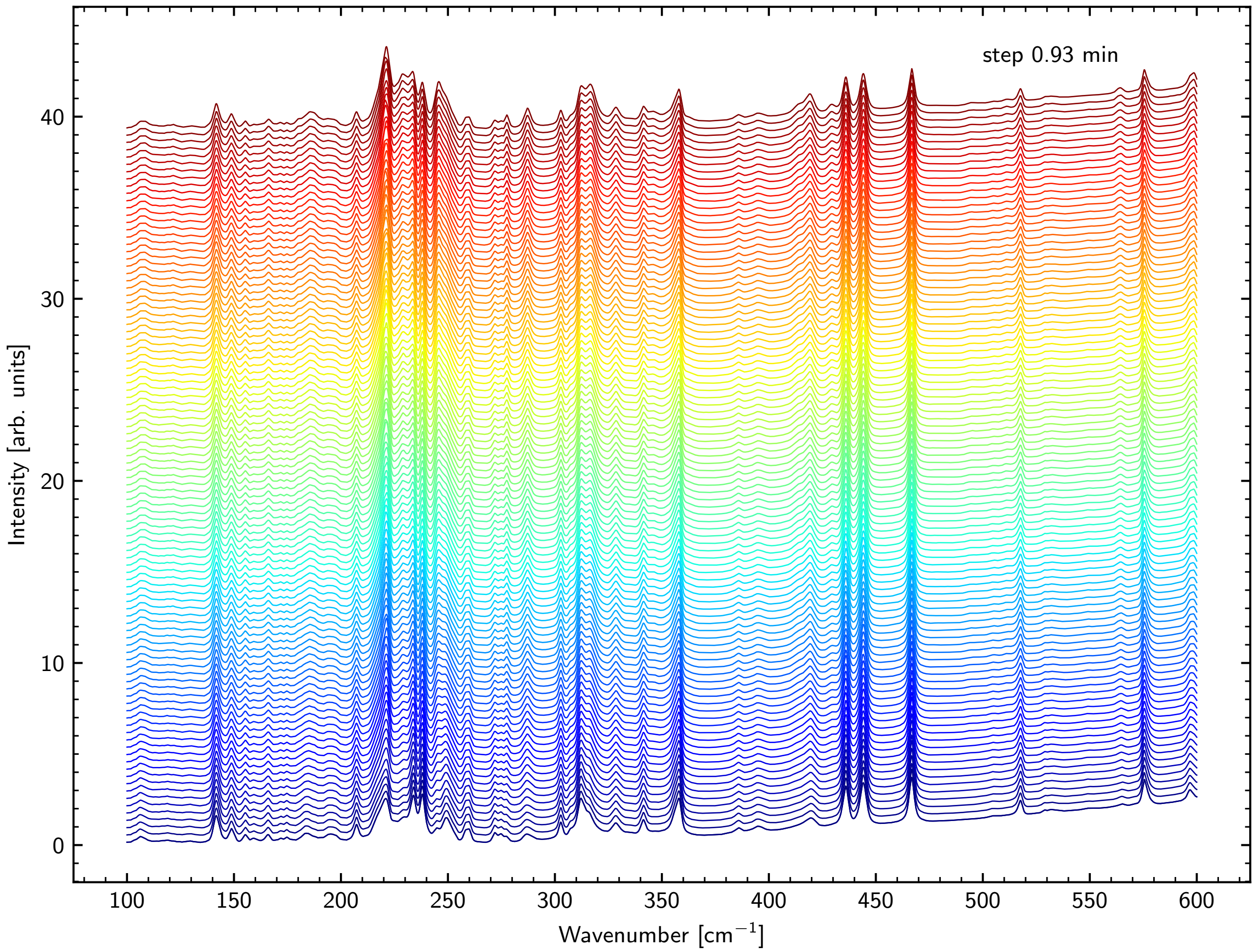

Figure S4: FIR spectra of the dinculear complex (**2**) in absorbance as a function of illumination time with 520 nm green laser.

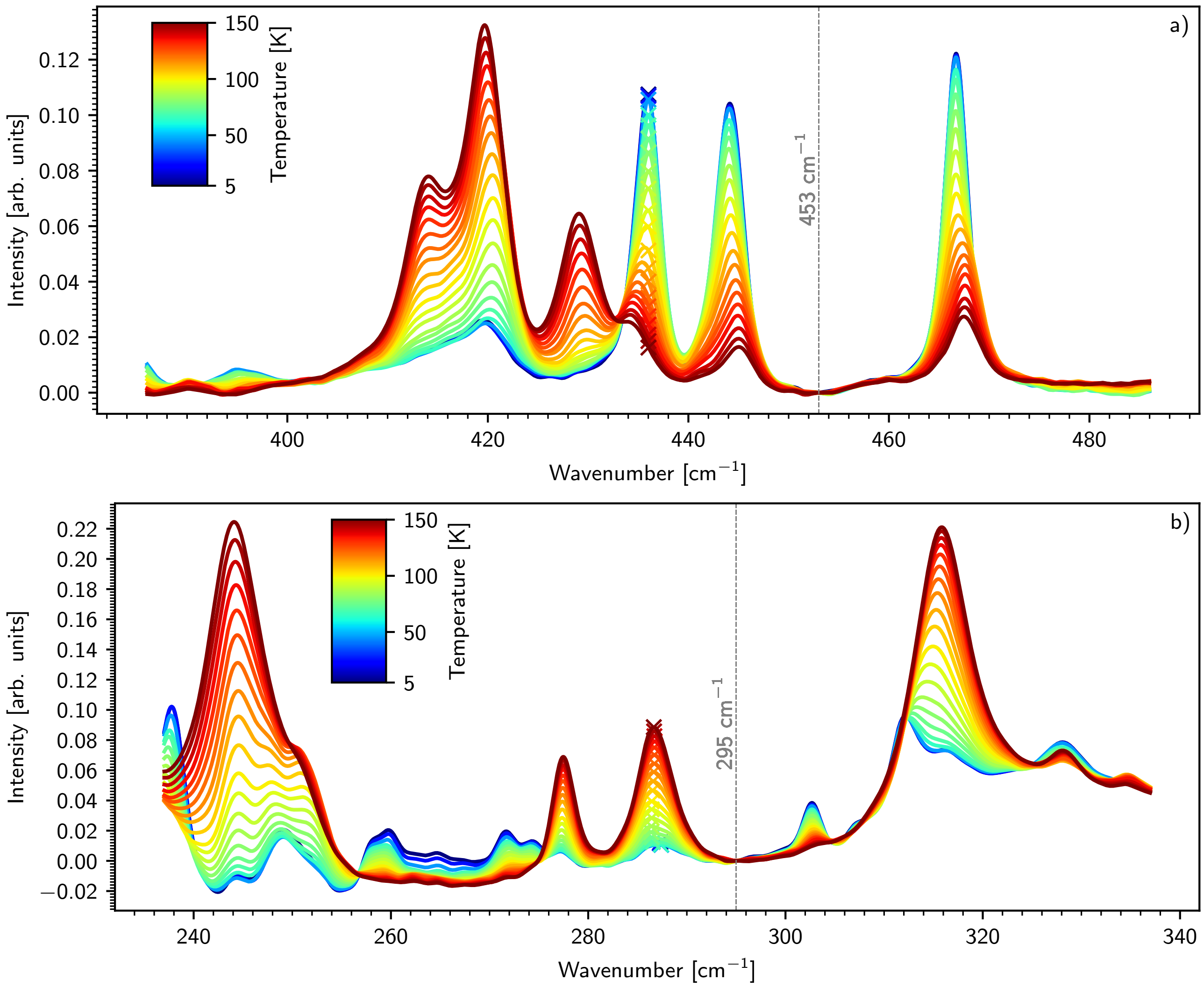

Figure S5: Baseline correction for the cooling cycle of the dinuclear complex (**2**) for the peaks ; H and J a) and E b) as shown in Figure 5. The vertical grey dashed line indicates the position of the baseline correction.

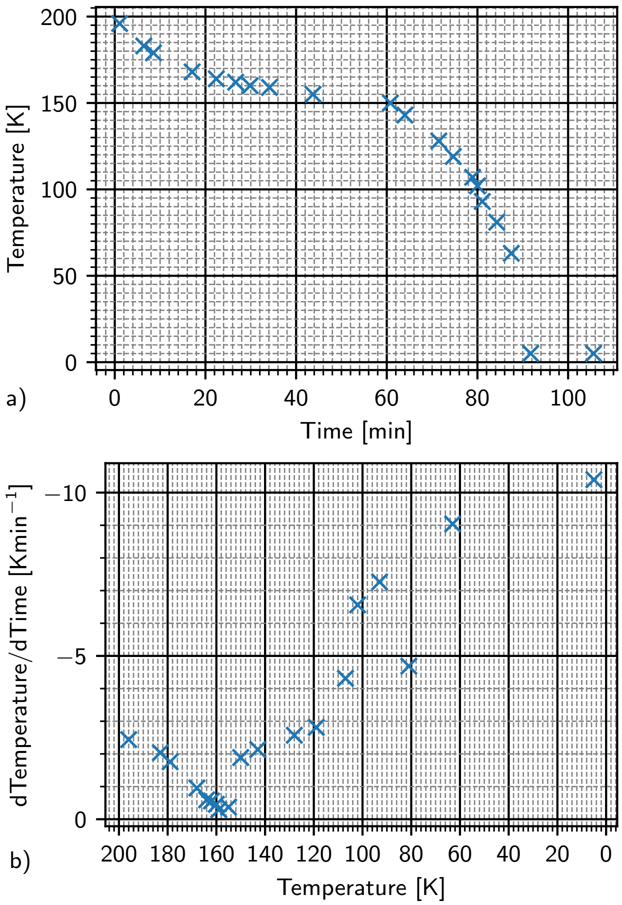

Figure S6: (a) Temperature over time and (b) cooling rate for the measurements on the mononuclear complex (**1**) shown in Figure 3.

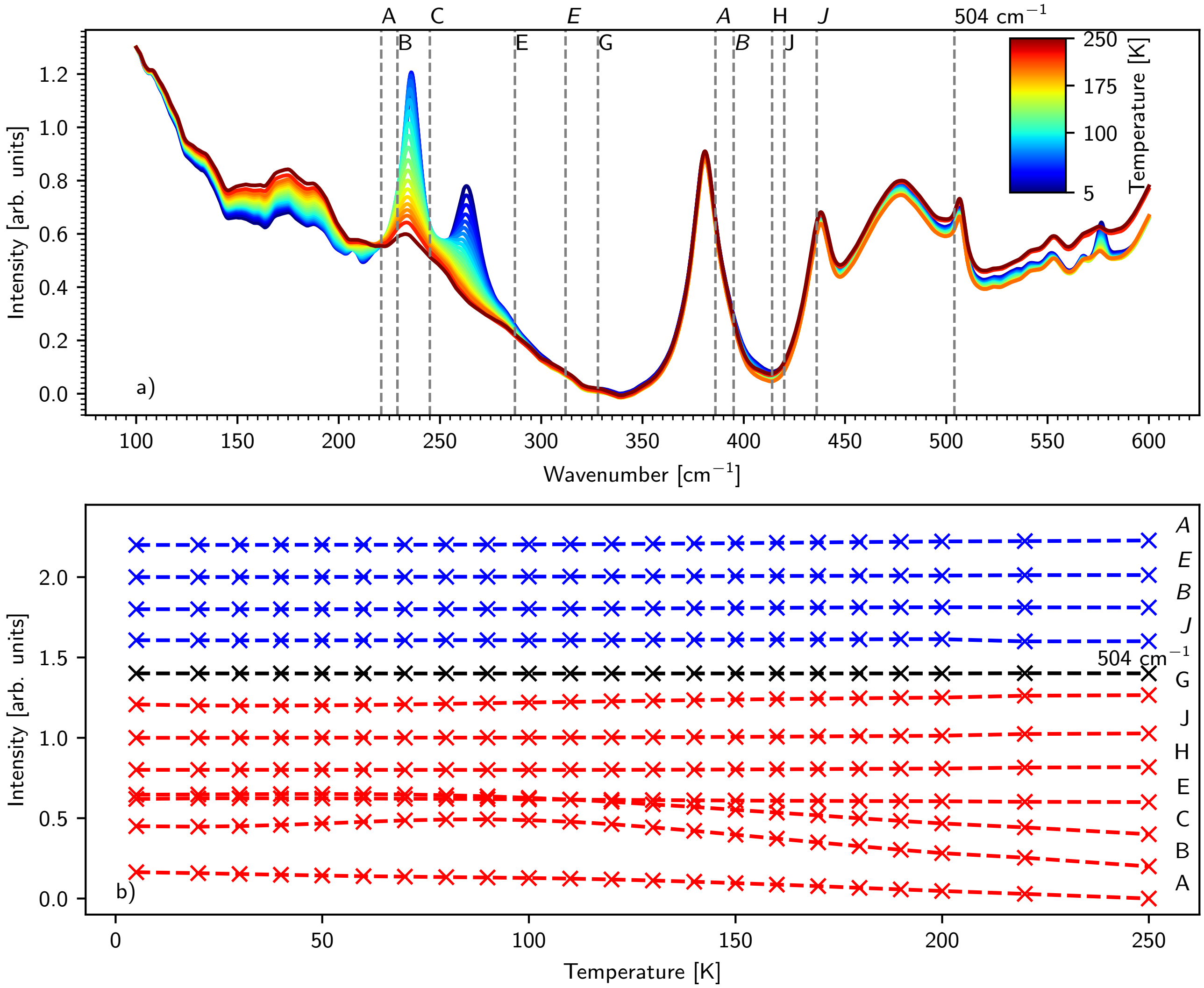

Figure S7: (a) Temperature dependence FIR absorption of HDPE and (b) the peak intensities at selected wavenumbers. For comparison, the naming of wavenumbers is adopted from Figure 5a.

Table S4: Vibrational modes of the HS-LS state of the dinuclear complex (**2**): experimental data and calculations (Orca 6.1-TPSSh-def2tzvp).

	HS-Fe* [cm^{-1}]		LS-Fe* [cm^{-1}]		Shifts (LS-HS) [cm^{-1}]	
Mode	assigned (± 0.2 cm^{-1})	calculated	assigned (± 0.2 cm^{-1})	calculated	assigned (± 0.2 cm^{-1})	calculated
Fe_{yz} (A)	221	230	386	410	165	180
Fe_{zy} (B)	229	219	395	392	166	173
Fe_{x} (C)	245	264	419	413	175	149
Bpz_1 (D)	278	275	287	309	9	34
Bpz_2 (E)	287	280	312	310	25	30
Bpz_3 (F)	315	317	328	322	13	5
$BipyAc_{str}$ (G)**	**335**	339	**335**	339	- -	- -
$Bipy_{bend}$ (H)**	414	429	414	429	- -	- -
$Bipy_{bend}$ (I)**	429	467	429	467	- -	- -
$Ac_{in\ plane}$ (J)**	434	432	434	432	- -	- -
$Bipyacbipy_{twist}$ -x-$ac_{in\ plane}$ **	**504**	518	**504**	518	- -	- -

* Vibrational modes exhibit small fractions of combined modes but consist predominantly of the indicated vibrational mode.

** Vibrational modes do not split into two sets of modes within the same molecule and are thus found only once.

Table S5: Calculated Fe-N distances of the different spin states (in Angström).

	HS-HS	HS-LS	LS-LS
$Fe-N_{bipy}$	2.166	2.163-1.949	1.949
$Fe-N_{bipy}$	2.167	2.169-1.948	1.948
$Fe-N_{bpz}$	2.129	2.129-1.988	1.989
$Fe-N_{bpz}$	2.132	2.134-1.988	1.988
$Fe-N_{bpz}$	2.189	2.190-2.000	2.000
$Fe-N_{bpz}$	2.192	2.194-2.001	2.001

Table S6: Calculated thermochemistry of the different spin states at 125 K (in kJ/mol). Please note: the absolute values of E_{el}, E_{el}+ZPE, H(corrected) and G_{final} deviate from the 4th digit onward, so for clarity reasons the presented values are shown only from the 4th digit on. The calculated absolute values are obtained by adding -14400000 to each presented value for the energies mentioned (eg.: $E_{el,calc}$=-51686.25-14400000=-14451686.25).

	**HS-HS**	**HS-LS**	**LS-LS**	**Δ** HS-HS - LS-LS	**M** (LS-LS+Δ/2)	**W** ((HS-LS)-M)
E_{el}	-51686.25	-51739.11	-51792.16	105.91	-51739.20	0.097
ZPE	2351.61	2363.60	2375.69	-24.08	2363.65	-0.053
$Corr_{vib}$	33.77	30.96	28.17	5.60	30.97	-0.015
$Corr_{rot}$	1.56	1.56	1.56	0	1.56	0
$Corr_{trans}$	1.56	1.56	1.56	0	1.56	0
E_{el}+ZPE	-49334.64	-49375.51	-49416.47	81.83	-49375.56	0.044
H (corr.)	-49296.72	-49340.40	-49384.14	87.43	-49340.43	0.028
S_{el}	2.28	1.67	0.00	2.284	1.142	0.531
S_{vib}	60.76	55.33	50.32	10.44	55.54	-0.206
S_{rot}	20.506	20.455	20.399	0.107	20.453	0.003
S_{trans}	22.156	22.156	22.156	0	22.156	0
S_{final}	105.704	99.617	92.876	12.828	99.290	0.327
G_{final}	-49402.42	-49440.02	-49477.02	74.60	-49439.72	-0.299

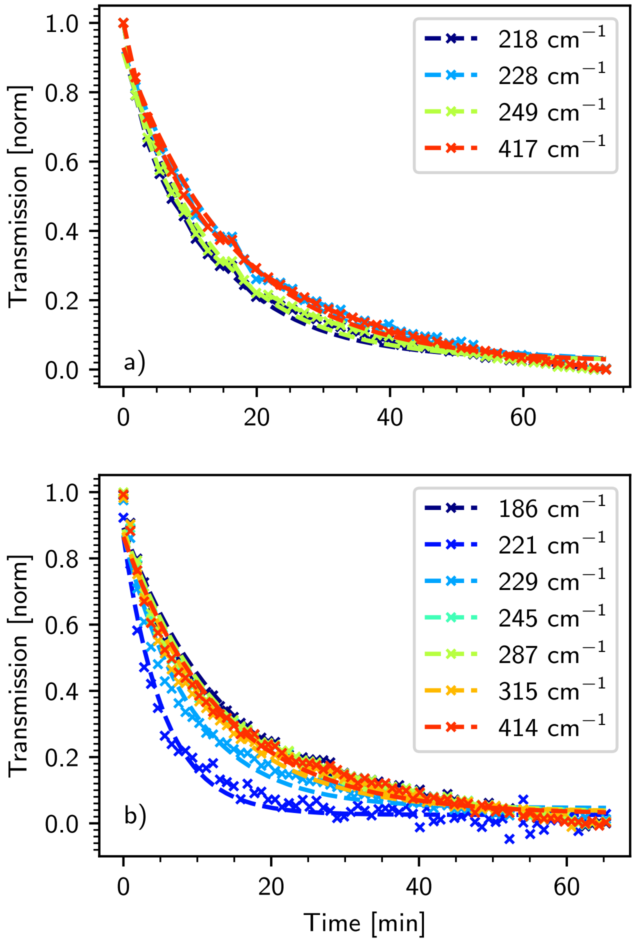

Figure S8: The intensity change of the modes after switching on the light as a function of time at 5 K (a) the mononuclear complex (**1**), (b) the dinuclear complex (**2**). Dotted lines show the exponential fits to the normalized transmission intensity. The obtained parameters are given in Table S3 for the mononuclear (**1**) and the dinuclear complex (**2**), respectively.

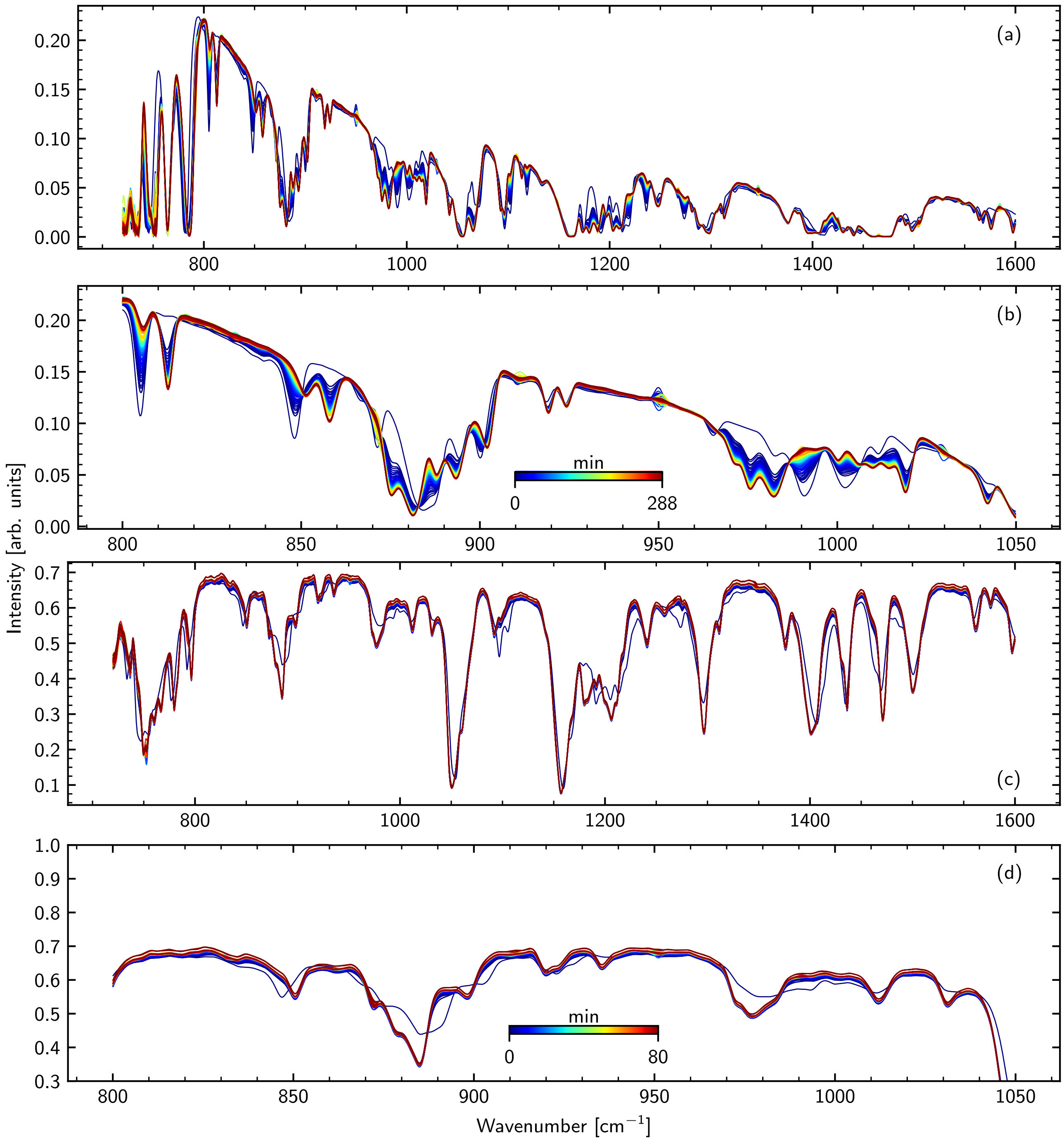

Figure S9: MIR transmittance spectra of (a) the mononuclear complex (**1**) (c) dinuclear complex (**2**) color-coded as a function of exposure time with a 520 nm green laser, with corresponding closeups in (b) and (d) respectively, measured at 5 K. [The slight offset at some peaks in (a) is given by the uncertainty of the amount of PE which shifts the baseline a bit in negative direction.]

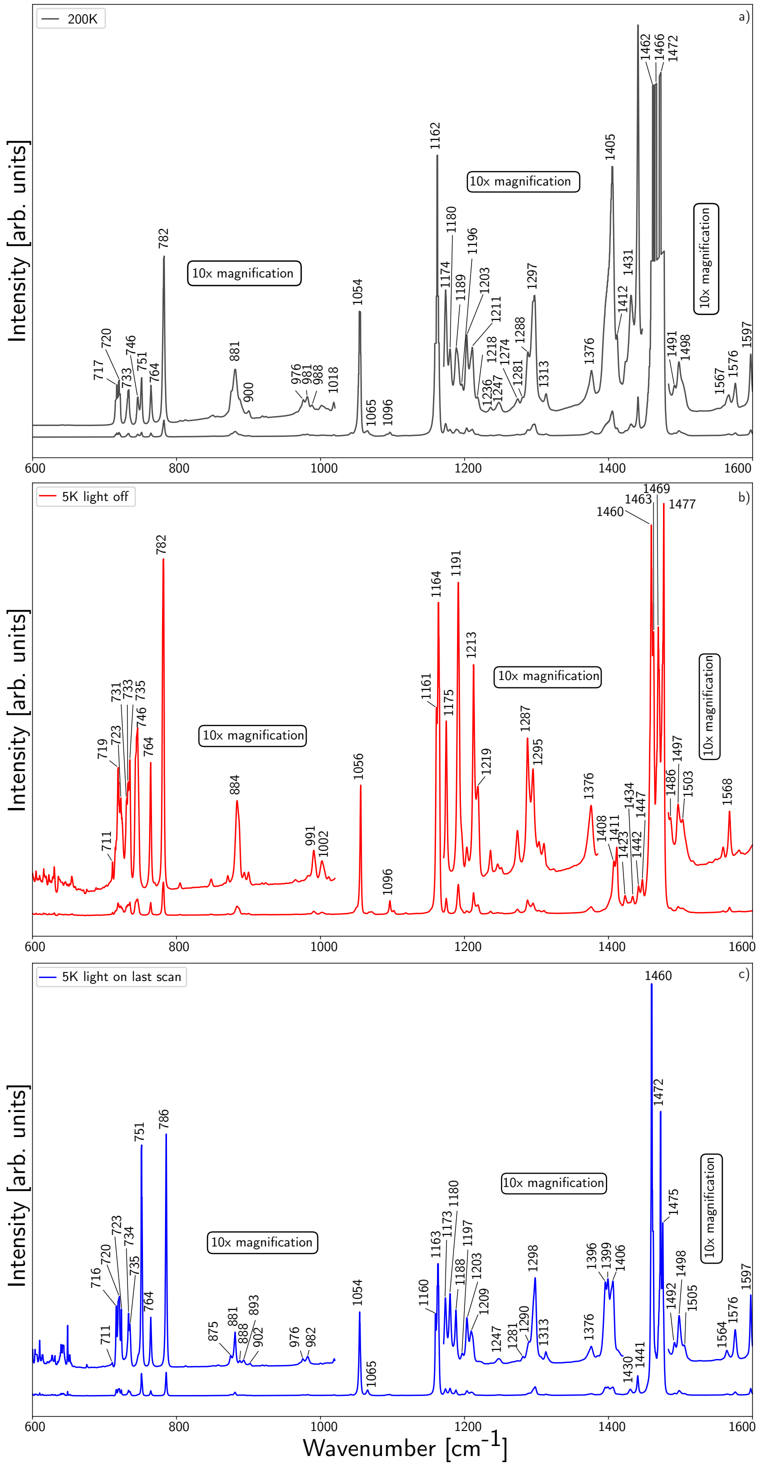

Figure S10: MIR absorbance spectra taken for the mononuclear complex (**1**) at 200 K (a), 5 K with light on (b) and 5 K (c). The spectrum with light exposure is the last scan after 80 min. of light exposure.

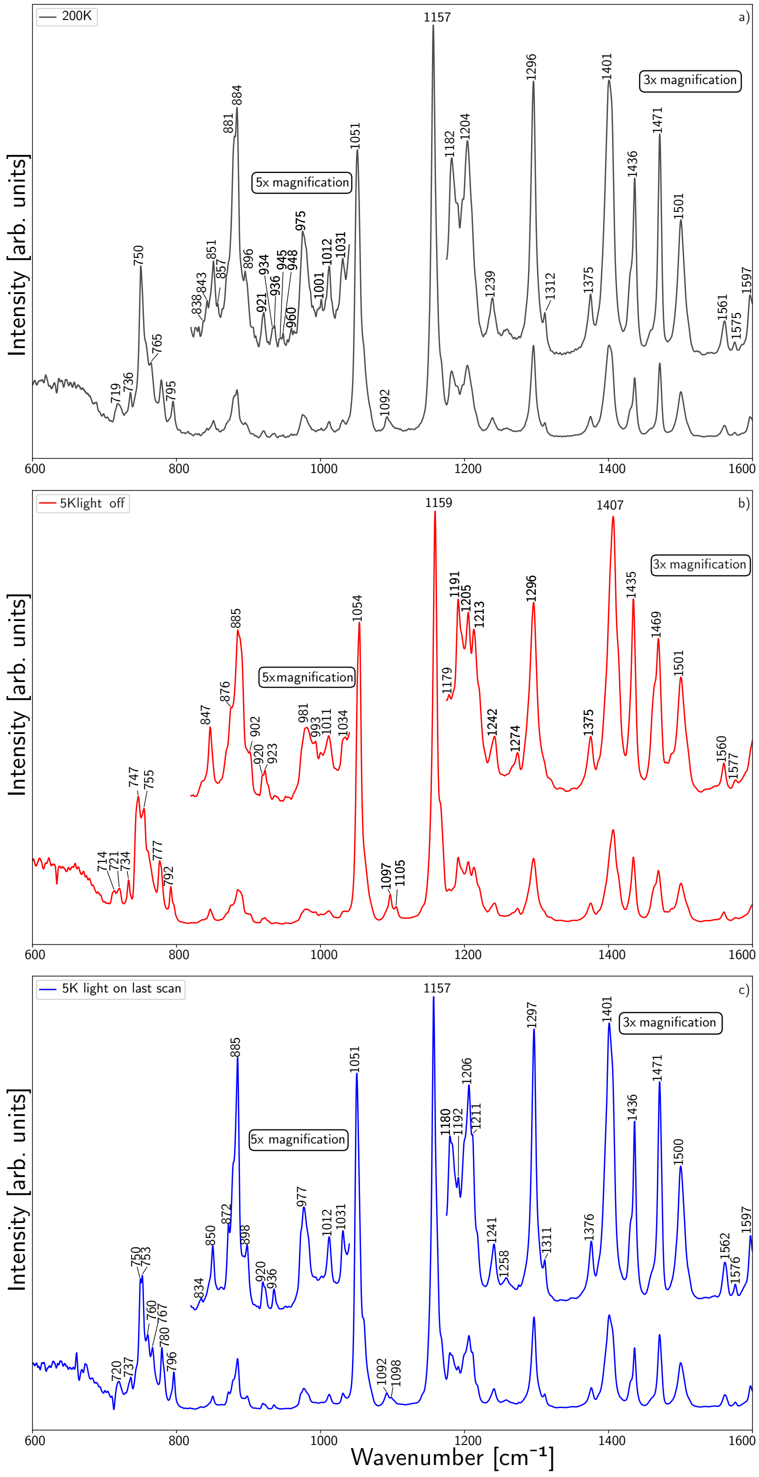

Figure S11: MIR absorbance spectra taken for the dinuclear complex (**2**) at 200 K (a), 5 K with light on (b) and 5 K (c). The spectrum with light exposure is the last scan after 80 min. of light exposure.

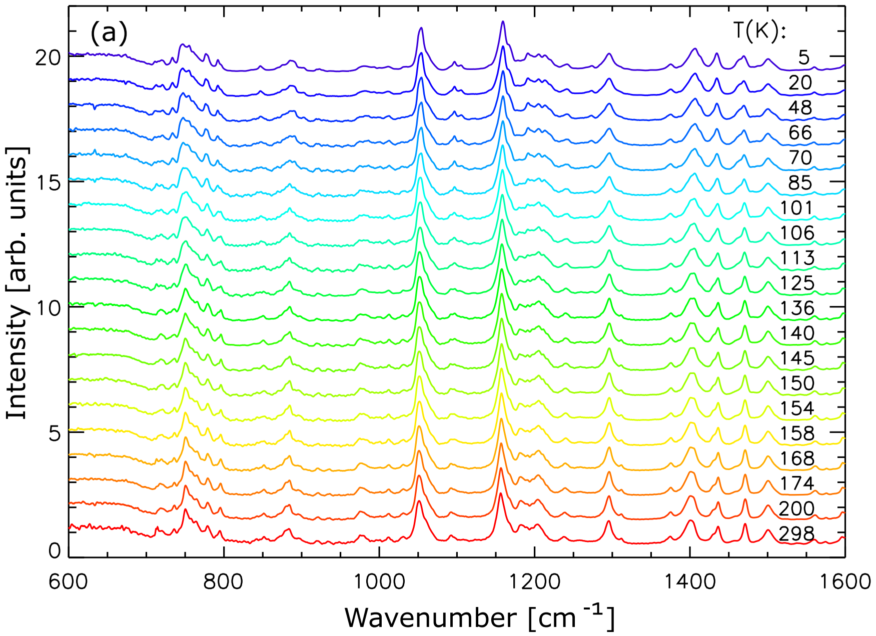

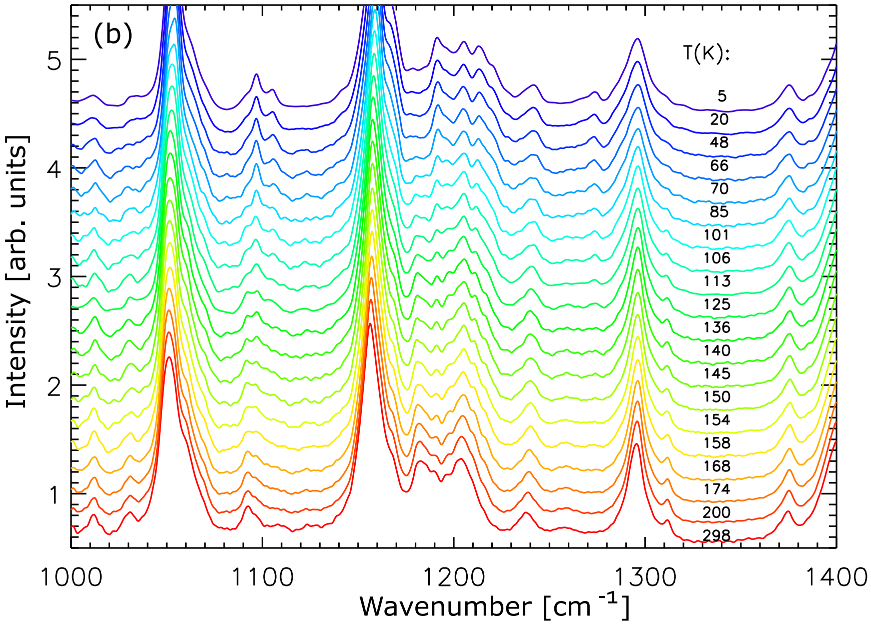

Figure S12: a) Absorbance spectra of the dinuclear complex (**2**) in the MIR range with temperature dependence. b) A closeup of absorbance spectra of the dinuclear complex (**2**) in the MIR range with temperature dependence.

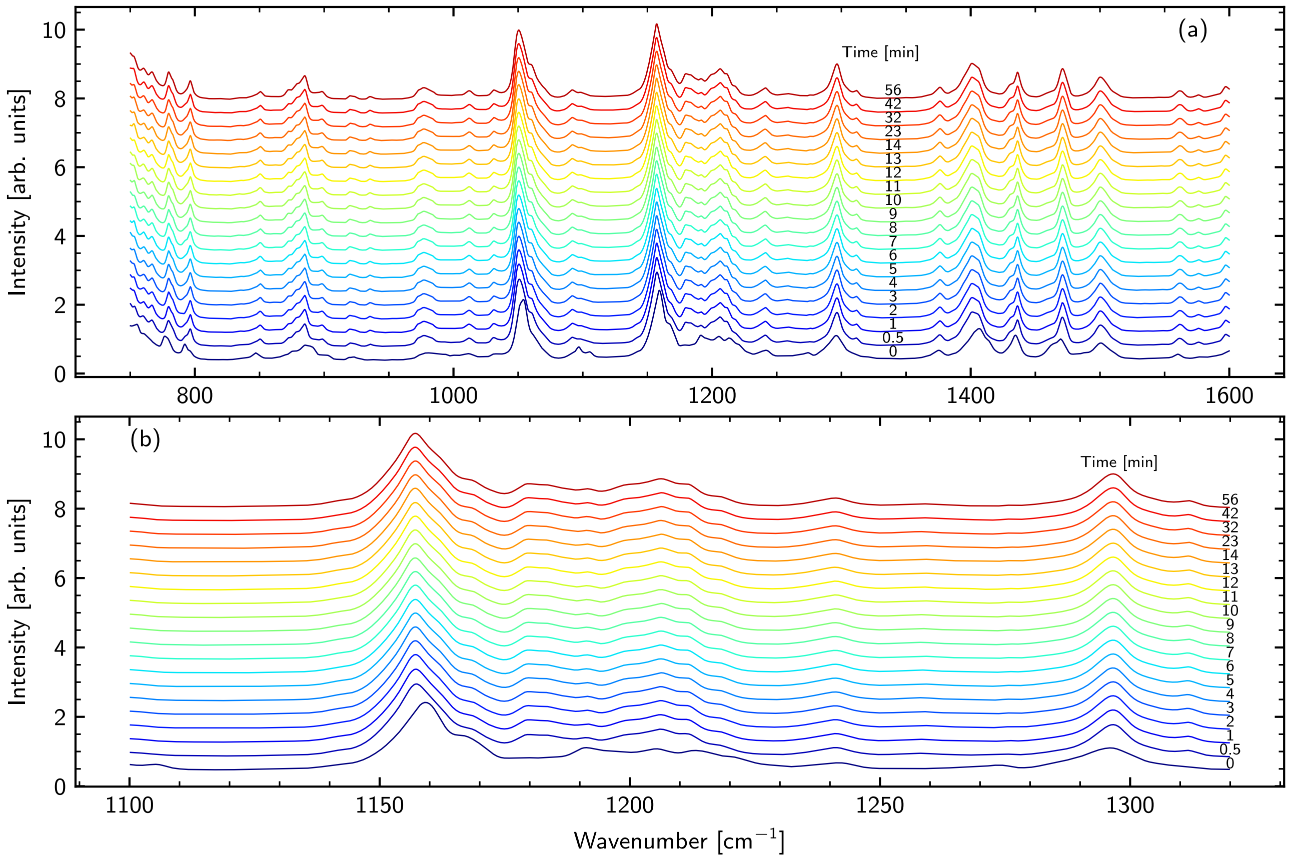

Figure S13: Absorbance spectra of the dinuclear complex (**2**) in the MIR range recorded at 5 K as a function of light illumination time. (a) fingerprint range, (b) close up

References

(1) Ossinger, S.; Naggert, H.; Bill, E.; Näther, C.; Tuczek, F. Electronic Structure, Vibrational Spectra, and Spin-Crossover Properties of Vacuum-Evaporable Iron(II) Bis(Dihydrobis(Pyrazolyl)Borate) Complexes with Diimine Coligands. Origin of Giant Raman Features. *Inorganic Chemistry* **2019**, *58*, 12873–12887, PMID: 31525895.
